# Processing-Tuned Laser-Induced Graphene Transducers for Polyaniline pH Sensors

**DOI:** 10.1021/acsomega.6c09091

**Published:** 2026-10-27

**Authors:** Atik Saha, Bo Mi Lee

**Affiliations:** Department of Mechanical and Aerospace Engineering, 14717 Missouri University of Science and Technology, Rolla, Missouri 65409, United States

## Abstract

Flexible pH sensors are important for wearable, biomedical, environmental, and process-monitoring applications, but achieving simple, scalable fabrication while maintaining high sensing performance remains challenging. Laser-induced graphene (LIG) has emerged as a promising transducer material because it enables direct laser patterning of porous, conductive graphitized carbon electrodes on flexible substrates. Although laser-processing conditions are known to affect LIG morphology, graphitization, and electrical properties, their influence on pH-sensitive layer formation and pH-sensing performance has not been systematically investigated. In this study, polyaniline (PANi)-modified LIG pH sensors were fabricated using four laser-processing combinations of laser power and scan speed. The resulting LIG and PANi/LIG electrodes were characterized using sheet-resistance measurements, optical microscopy, SEM, Raman spectroscopy, and XPS. PANi was electropolymerized on the LIG working electrodes under identical cyclic voltammetry conditions, and the assembled sensors were evaluated in pH 4–9 buffer solutions. The results showed that laser processing produced distinct LIG morphologies, porosities, sheet resistance, and PANi coverage, leading to different sensing performances. Among the samples, PANi/LIG-9.2–350, fabricated at 9.2 W and 350 mm/s, exhibited the best overall performance, with a near-Nernstian sensitivity of −60.04 mV/pH, a 12 h drift rate of 1.67 mV/h, hysteresis of 2.06 mV, and rapid response time of 13.2 s. These findings emphasize the significance of laser-processing-dependent LIG microstructure and electrical properties in governing PANi deposition behavior and resulting potentiometric pH-sensing performance.

## Graphical abstract

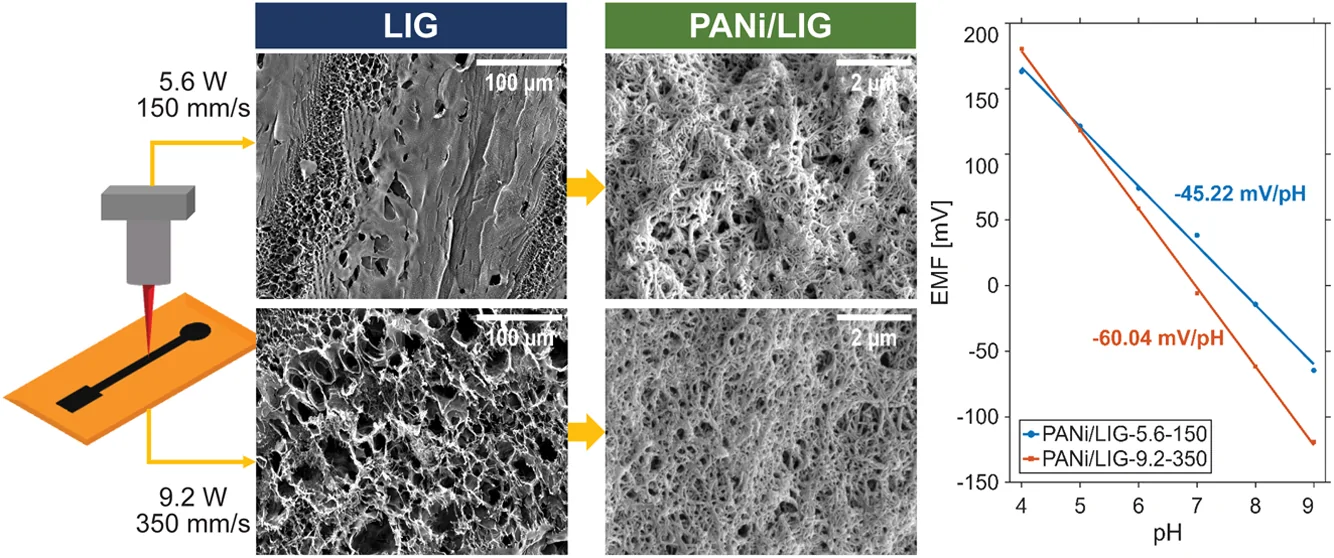

## 1 Introduction

Flexible pH sensors are increasingly important for wearable health monitoring, biomedical diagnostics, and environmental monitoring because they enable measurement of local hydrogen-ion activity through conformal contact with curved, irregular, or soft surfaces.^1–3^ In flexible pH sensors, materials typically perform one or more functional roles: pH-sensitive recognition, electrical transduction, or both. pH-responsive materials such as polyaniline (PANi), polypyrrole, and polydopamine can generate pH-dependent electrical responses through protonation/deprotonation or other ion-sensitive interfacial reactions.^4,5^ In contrast, electrically conductive materials such as printed carbon inks, silver inks, carbon nanotubes (CNT), and MXenes can serve primarily as transducing platforms by providing conductive pathways and electrical interfaces for sensing signal transduction.^6–10^ In some sensor architectures, a single material can serve both sensing and transducing functions. For instance, conductive metal oxides such as RuO*_x_* can generate a pH-dependent electrode potential through proton-coupled redox equilibria between different metal oxidation states while simultaneously serving as the conductive electrode.^11^

Among electrically conductive transducing materials, carbon-based nanomaterials such as CNTs and mesoporous carbon have attracted significant attention because of their high electrical conductivity, large specific surface area, and chemical stability.^7,12–14^ However, fabrication and patterning of conventional carbon-based electrodes often require photolithography or template-based printing processes that increase fabrication complexity and limit rapid design modification.^8,15–17^ Laser-induced graphene (LIG) has emerged to address these limitations, offering a low-cost, binder-free, scalable, and one-step fabrication approach.^18–20^ Laser irradiation locally converts the polymer surface into a porous and electrically conductive sp^2^-rich graphitic carbon network, enabling the substrate to function simultaneously as a flexible support and a patterned transducing electrode.^21–25^ LIG has therefore been combined with pH-responsive materials, including PANi, poly­(3,4-ethylenedioxythiophene):poly­(styrenesulfonate) (PEDOT:PSS), and polyfolate (PFA) to provide proton-sensitive functionality.^25–27^ Among these materials, PANi is particularly attractive because of its simple synthesis, environmental stability, mechanical flexibility, and reversible protonation/deprotonation in response to H^+^ activity. Recent studies have demonstrated the integration of PANi/LIG pH sensors into flexible and wearable platforms, including multifunctional smart bandages for wound monitoring and drug delivery, diaper-integrated sensors for urine analysis, and multimodal wound-sensing systems for simultaneous biomarker detection.^23,26,28^ However, these studies primarily focused on sensor performance and device-level applications rather than systematically examining how laser-processing-dependent LIG properties influence PANi deposition and the resulting pH-sensing behavior. In fact, laser-processing parameters such as laser power, scan speed, focal distance, and pulse density are known to alter LIG morphology, surface area, and electrical properties.^18,19,29^ For example, Behrent et al. demonstrated that variations in laser power, scanning speed, and pulse density altered LIG morphology and electrical resistance, resulting in varied electrochemical sensing performance,^18^ while Bhattacharya et al. showed that laser irradiation conditions modified the microstructure and electrochemical surface area of LIG and consequently affected uric-acid sensing.^30^ Thus, although PANi/LIG pH-sensing and laser-processing-dependent changes in LIG properties have each been demonstrated, the process-structure-performance relationships linking laser-processing-dependent LIG transducer properties to PANi deposition and structure, and ultimately to potentiometric pH-sensing performance have not been systematically investigated.

In this study, we systematically evaluated the process-structure–property relationship of PANi-modified LIG pH sensors. Specifically, we investigated how different combinations of laser-processing parameters (i.e., laser power and scan speed) affect the morphology, surface area, and electrical properties of LIG; how these laser-processing-dependent LIG properties influence the morphology and physicochemical structure of electropolymerized PANi; and how the resulting PANi/LIG electrode structure affects potentiometric pH-sensing performance. We hypothesized that laser-processing-dependent LIG morphology and electrical properties affect pH-sensitive PANi deposition, thereby leading to variations in pH-sensing behavior. To test this hypothesis, four representative laser-processing conditions were selected from the viable processing window based on initial optical microscopy observations and electrical property screening. The selected LIG electrodes were compared in terms of morphology, apparent porosity, sheet resistance, specific surface area, and graphitic structure, and subsequently used as transducing layers for PANi electropolymerization. The resulting PANi/LIG electrodes were further characterized for PANi morphology, elemental distribution, surface chemical composition, and structural characteristics, followed by potentiometric evaluation of pH-sensing performance, including sensitivity, 12 h drift stability, reversibility, repeatability, and response time. The overall best-performing PANi/LIG sensor combined low sheet resistance, high apparent porosity, high specific surface area, a continuous conductive carbon network, and uniform PANi coverage, which together likely contribute to a greater electrolyte-accessible interfacial area and a more homogeneous proton-responsive PANi layer. By correlating these laser-processing-dependent LIG properties with PANi deposition behavior and pH-sensing behavior, we show that LIG transducer characteristics strongly influence the formation of the PANi layer and the resulting potentiometric pH-sensing performance, emphasizing the importance of understanding the LIG process-structure–property relationship in PANi/LIG pH sensor design. More broadly, advances in flexible sensing materials can complement emerging intelligent healthcare and rehabilitation technologies, in which sensing is increasingly integrated with computational approaches.^31,32^

## 2 Materials and Experimental Methods

### 2.1 Materials

Polyimide (PI) film with a thickness of 127 μm was purchased from McMaster-Carr (USA). Aniline, polyvinyl butyral (PVB), potassium chloride (KCl), sodium hydroxide (NaOH), and methanol were purchased from Sigma-Aldrich (USA). Isopropyl alcohol (IPA) was obtained from Florida Laboratories Inc. (USA). Ag/AgCl ink was purchased from Creative Materials (USA). Hydrochloric acid was acquired from Thermo Fisher Scientific (USA). All chemicals were of analytical grade and used without any further treatment.

### 2.2 LIG Fabrication and Process Parameter Selection

LIG was fabricated on 127 μm-thick PI film using a computer-controlled CO_2_ laser platform (Fusion Edge 12, 10.6 μm, Epilog Laser, USA). A broad range of laser powers (2–10.4 W) and scan speeds (30–400 mm/s) was initially explored to establish a processing window to evaluate their influence on LIG formation. All LIG patterns were fabricated in raster mode under ambient conditions, with the designed areas filled line-by-line at a fixed raster density of 500 DPI. The resulting LIG samples were visually classified into three categories: unconverted, viable, and burnt (Figure 1). Unconverted samples showed little or no visible carbonization of the PI surface, whereas viable samples exhibited a continuous carbonized pattern without PI surface damage, and burnt samples showed excessive laser-ablation and physical damage of the PI film due to excessive thermal energy input. Only viable LIG samples were selected for further evaluation. To identify representative processing conditions, the viable LIG samples were compared based on their electrical properties and optically observed surface morphology. Optical microscopy images were obtained using a digital microscope (Hirox, USA) for preliminary qualitative assessment of the LIG surface morphology. Optical microscopy revealed visible differences in surface texture and apparent porous features among the viable LIG samples, and the samples were qualitatively categorized into low-, medium-, and high-porosity groups.

**Figure 1:**
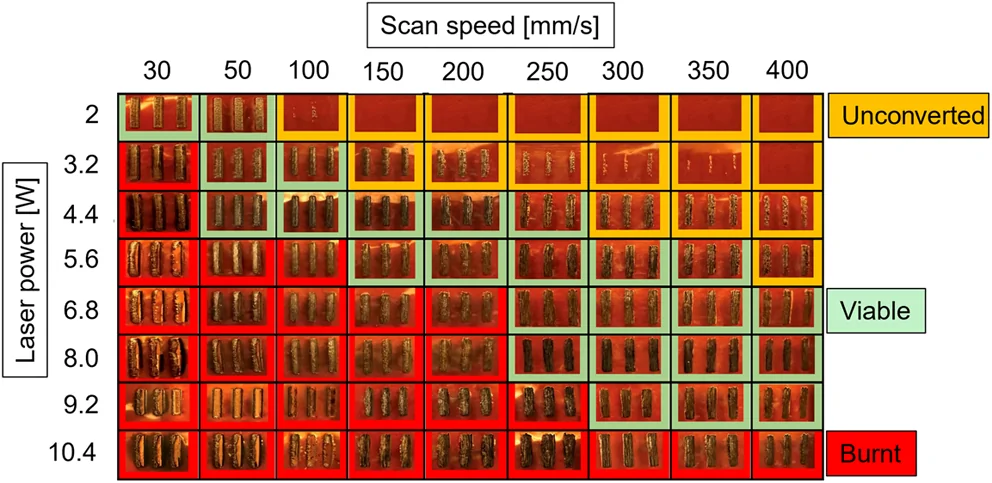
LIG formation at varying laser powers and scan speeds, visually classified as unconverted, viable, or burnt.

### 2.3 Electrical Characterization of LIG

The electrical properties of the LIG electrodes were evaluated by sheet resistance measurements using a four-point probe (Ossila BV, Netherlands). Sheet resistance was selected as the electrical property metric because LIG forms a laterally conductive carbon layer with nonuniform thickness on the PI substrate, making bulk resistivity difficult to determine. Four collinear probes were placed in contact with the LIG surface, with current applied through the two outer probes and the resulting voltage difference measured between the two inner probes. This configuration minimizes contributions from probe-contact and lead resistance to the measured voltage. Sheet resistance was calculated from the measured voltage-to-current ratio using the appropriate geometric correction factor implemented in the Ossila Sheet Resistance Lite software. Three samples from each laser-processing condition were measured, and the mean sheet resistance was calculated. The sheet resistance values were then classified into relatively low, intermediate, and relatively high sheet resistance categories to compare the effects of different laser-processing conditions.

### 2.4 Fabrication of PANi-Based pH Sensor

The fabrication of the pH sensor began with the formation of LIG electrodes on PI film using the predetermined laser power and scan speed described in Section 2.2 (Figure 2). The laser-patterned LIG electrode consisted of two components: a reference electrode and a working electrode. The working electrode was designed as a circular pad with a radius of 1.5 mm, while the reference electrode was designed as an arc-shaped region bounded by inner and outer radii of 2.5 and 3.5 mm, respectively. The reference electrode region was printed with Ag/AgCl ink using a V-One printer (Voltera, Canada) at a printing speed of 400 mm/min, followed by drying at 100 °C for 5 min. Then, 2 μL of PVB solution (10 wt % in methanol) containing KCl powder (30% w/v) was drop-cast onto the Ag/AgCl layer using a micropipette. The reference electrode area was conditioned by immersion in 3 M KCl for 24 h. The working electrode was modified with PANi via electropolymerization using cyclic voltammetry (CV) with a potentiostat (1010E, Gamry Industries, USA). The LIG electrode was immersed in an aqueous solution containing 1 M HCl and 0.1 M aniline, and CV was performed over a potential range of −0.2 to +1.0 V for 30 cycles at a scan rate of 50 mV s^–1^. The consistent CV conditions were used throughout the samples since the focus of this study was to investigate the effects of LIG layers on the pH-sensing behaviors. Following electropolymerization, the electrode was dried at 40 °C for 8 min, resulting in the formation of a visible green PANi coating on the working electrode surface (Figure 2f).

**Figure 2:**
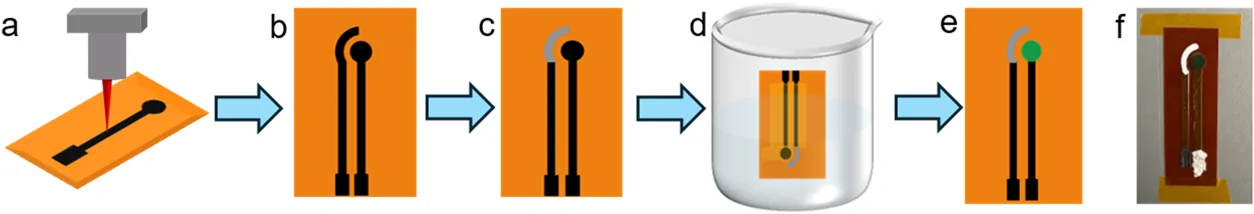
Schematic illustration of the pH sensor fabrication process: (a) LIG electrode formation on a PI film; (b) patterned LIG tracks; (c) Ag/AgCl ink deposition; (d) reference electrode conditioning in 3 M KCl; (e) completed PANi/LIG pH sensor; and (f) photograph of the fabricated sensor.

### 2.5 Morphological and Physicochemical Characterization for LIG and PANi/LIG Electrodes

The morphological and physicochemical characteristics of the selected LIG and PANi/LIG electrodes were evaluated using optical microscopy, SEM, EDS, Raman spectroscopy, and XPS. Raman spectra of the LIG and PANi/LIG electrodes were obtained using a Raman spectrometer (iXR, Thermo Scientific) with a 532 nm excitation laser at the power of 2.0 mW and 0.5 mW, respectively, and an exposure time of 5 s. To evaluate variations in surface morphology, SEM imaging was performed using a Prisma E variable-pressure scanning electron microscope (Thermo Fisher Scientific, USA) in secondary electron mode with an Everhart-Thornley detector (ETD). Prior to SEM imaging, the samples were sputter-coated with Au/Pd for 5 min. Energy dispersive X-ray spectroscopy (EDS) was obtained using a Helios 5 Hydra CX SEM. Broad-scan X-ray photoelectron spectroscopy (XPS, NEXSA, Thermo Fisher Scientific, USA) was performed using a monochromated Al­(Kα) X-ray source operating at 1486.6 eV and an X-ray spot size of 330 μm to evaluate changes in surface elemental composition after PANi electropolymerization.

### 2.6 pH-Sensing Characterization

The pH-sensing performance of the PANi/LIG pH sensors was evaluated by measuring the electromotive force (EMF) response in buffer solutions over a pH range of 4 to 9. EMF measurements were carried out under open-circuit conditions using a potentiostat.^33^ Prior to the electrochemical measurements, the pH of each buffer solution was verified using a commercial pH meter (FiveGo F2, Mettler Toledo, Sweden). The PANi-modified LIG serves as a pH-sensitive working electrode, while the Ag/AgCl on LIG functions as the reference electrode. For each measurement, the sensor was immersed in the corresponding buffer solution, and the EMF response was recorded after signal stabilization.

## 3 Results and Discussion

### 3.1 Electrical Properties of LIG and Laser-Processing Parameter Selection

During the initial LIG screening process, the electrical properties of the LIG electrodes were evaluated by measuring their sheet resistance. The complete sheet-resistance data from the initial screening are summarized in Table S1 (Supporting Information). At a fixed laser power, increasing scan speed generally increased sheet resistance. For example, at 4.4 W, the sheet resistance increased from 1.52 Ω/sq at 30 mm/s to 8.73 Ω/sq at 300 mm/s, while at 6.8 W, it increased from 6.95 Ω/sq at 200 mm/s to 11.09 Ω/sq at 400 mm/s. At a fixed scan speed, increasing laser power generally tended to decrease sheet resistance; however, this trend was not monotonic across the entire processing window. The viable LIG electrodes exhibited sheet resistance values ranging from 2.59 to 11.09 Ω/sq, which are comparable to previously reported LIG samples.^34,35^ Based on the distribution of measured sheet resistance values, the viable LIG electrodes were classified into three groups: relatively low-resistance (<5.42 Ω/sq), intermediate resistance (5.42–8.26 Ω/sq), and relatively high-resistance (>8.26 Ω/sq). Optical microscopy was then performed to qualitatively evaluate the surface morphology of the viable LIG electrodes. The electrodes were qualitatively classified as low-porosity, medium-porosity, or high-porosity groups based on the visible degree of pore formation. By correlating sheet resistance with the optically observed morphology, the LIG electrodes were further classified into four different categories: relatively low resistance-low porosity, relatively low resistance-high porosity, intermediate resistance-low porosity, and intermediate resistance-high porosity. These categories were selected to compare LIG electrodes with distinct combinations of sheet resistance and pore development for subsequent pH sensor fabrication. Relatively high-resistance samples were excluded from further analysis because they frequently exhibited poor adhesion and delamination, making them unsuitable for subsequent sensor fabrication. One representative laser-processing condition was selected from each category. The selected laser powers were 4.4, 5.6, 6.8, and 9.2 W, with corresponding scan speeds of 150, 150, 250, and 350 mm/s, respectively. The resulting LIG electrodes were labeled as LIG-4.4-150, LIG-5.6-150, LIG-6.8-250, and LIG-9.2-350, where the notation indicates laser power (W) followed by scan speed (mm/s). The selected LIG electrodes exhibited sheet resistance values in the range of 3.44–7.43 Ω/sq (Figure 3). Among the four selected processing conditions, LIG-4.4-150 and LIG-9.2-350 electrodes exhibited the lowest sheet resistance values of 3.44 Ω/sq, whereas LIG-5.6-150 and LIG-6.8-250 showed higher resistance of 7.43 Ω/sq and 7.12 Ω/sq, respectively. The variation in sheet resistance can be attributed to differences in laser energy input arising from the combined effects of laser power and scan speed. During CO_2_ laser irradiation, the energy input controls the degree of graphitization and the formation of continuous conductive pathways within the LIG network.^29,36^ To quantitatively compare the four selected conditions, we calculated the areal laser energy density, *E*_*A*_

1 $$E_{A}=\frac{P}{\mathrm{vh}}$$

where *P* is the laser power, *v* is the scan speed, and *h* is the spacing between adjacent raster lines.^37^ At 500 DPI, the raster-line spacing was 0.0508 mm. The nominal areal energy density results were 0.577, 0.735, 0.535, and 0.517 J/mm^2^ for LIG-4.4-150, LIG-5.6-150, LIG-6.8-250, and LIG-9.2-350, respectively. No monotonic relationship was observed between the calculated energy input and either sheet resistance or apparent porosity within these four LIG conditions. These results indicate that collapsing laser power and scan speed into a single nominal areal energy density does not fully describe LIG formation. Even at similar nominal energy inputs, different combinations of laser power and scan speed can produce different irradiation temperatures and dwell times, which can influence PI conversion and resulting LIG structure.^38,39^

**Figure 3:**
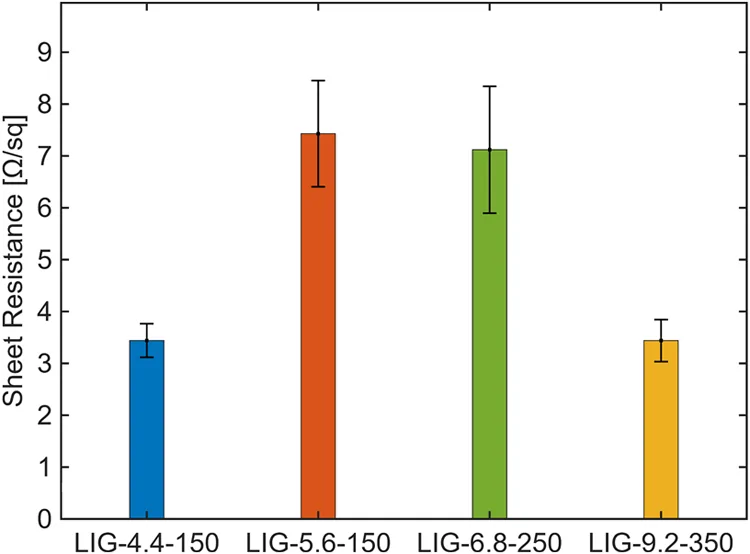
Sheet resistance of LIG electrodes fabricated using different laser-processing conditions. Error bars indicate the standard error of the mean.

Accordingly, the four selected processing conditions cannot be used to quantitatively separate the individual effects of laser power and scan speed because both parameters vary simultaneously for most of these conditions. Murray et al. showed that LIG sheet resistance depends on higher-order and interaction effects of laser power and scan speed, with distinct low-resistance regions occurring at different power-speed combinations rather than following a single monotonic relationship.^40^ Therefore, the differences in sheet resistance among the four selected processing conditions reflect the combined effects of laser power and scan speed rather than a simple dependence on either parameter individually. The resulting LIG morphology may also contribute to these differences: SEM observations indicated that LIG-6.8-250 exhibited discontinuities in the conductive network, whereas LIG-9.2-350 showed a more continuous and interconnected porous carbon structure.

### 3.2 Electropolymerization of PANi on LIG Electrodes

Electropolymerization of aniline to form PANi was performed by CV in an acidic electrolyte containing 0.1 M of aniline monomer and 1 M HCl (Figure S1, Supporting Information). Figure 4 shows the representative CV response of the LIG-4.4–150 electrode during PANi growth. In the initial cycles, a single anodic peak was observed at approximately +0.8 to +1.0 V (Figure S2, Supporting Information), which is attributed to the oxidation of aniline monomers to reactive cation-radicals.^41^ These cation-radicals subsequently undergo coupling reactions to form oligomers, initiating PANi nucleation and growth on the LIG surface. As cycling continued, the anodic response evolved, with peaks appearing near +0.2 to +0.3 V and later becoming more pronounced around +0.55 to +0.6 V (a in Figure 4). These peaks are associated with the characteristic oxidation transitions of PANi from leucoemeraldine base to emeraldine salt (ES) and from ES toward the pernigraniline base, respectively.^42^ During the reverse scan, a cathodic peak near +0.2 to +0.3 V (b in Figure 4) was observed, indicating reduction of oxidized PANi species.^43^ The progressive increase in current and peak intensity with successive CV cycles indicates continuous PANi deposition on the LIG surface, with the deposited PANi increasingly contributing to the observed redox response.

**Figure 4:**
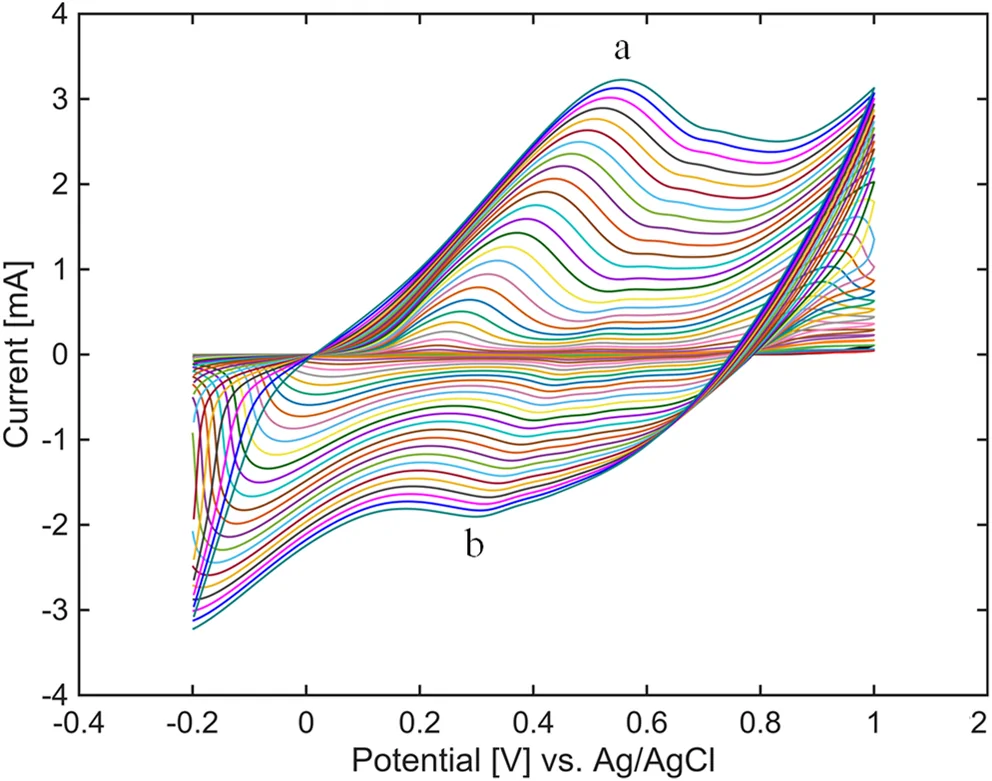
Cyclic voltammograms during electropolymerization of PANi on the LIG-4.4-150 electrode in 1 M HCl containing 0.1 M aniline.

To quantitatively compare the electrochemical response during PANi electropolymerization, the cumulative anodic charge was calculated from the CV data by integrating the anodic current over the forward sweep of each cycle and summing the charge over all 30 cycles.

The charge was calculated according to

2 $$Q=\sum_{n=1}^{30}\frac{1}{v}\int I\mathrm{dE}$$

where *I* is the anodic current during cycle *n* and *v* is the scan rate (0.05 V/s).

The cumulative anodic charges were 521.48, 486.06, 320.52, and 455.55 mC for LIG-4.4-150, LIG-5.6-150, LIG-6.8-250, and LIG-9.2-350, respectively. Because all four electrodes were electropolymerized under identical electropolymerization conditions, these differences indicate different electrochemical responses during PANi electropolymerization on the four LIG substrates. The cumulative anodic charge is not interpreted as a direct quantitative measure of PANi loading because the measured charge can include contributions from aniline monomer oxidation associated with polymerization initiation and repeated oxidation of previously deposited PANi.^44^

### 3.3 Morphological and Physicochemical Properties of LIG and PANi/LIG Electrodes

#### 3.3.1 Surface Morphology of LIG

The optical microscopy images of the selected LIG samples are shown in Figure 5a–d. LIG-4.4-150 and LIG-5.6-150 exhibited parallel line patterns corresponding to the raster scanning path, along with visible pores on the LIG surface (Figure 5a,b). LIG-6.8-250 and LIG-9.2-350 showed less visible parallel lines and more widely distributed pores on the LIG surface (Figure 5c,d), suggesting greater pore development under these laser-processing conditions.

**Figure 5:**
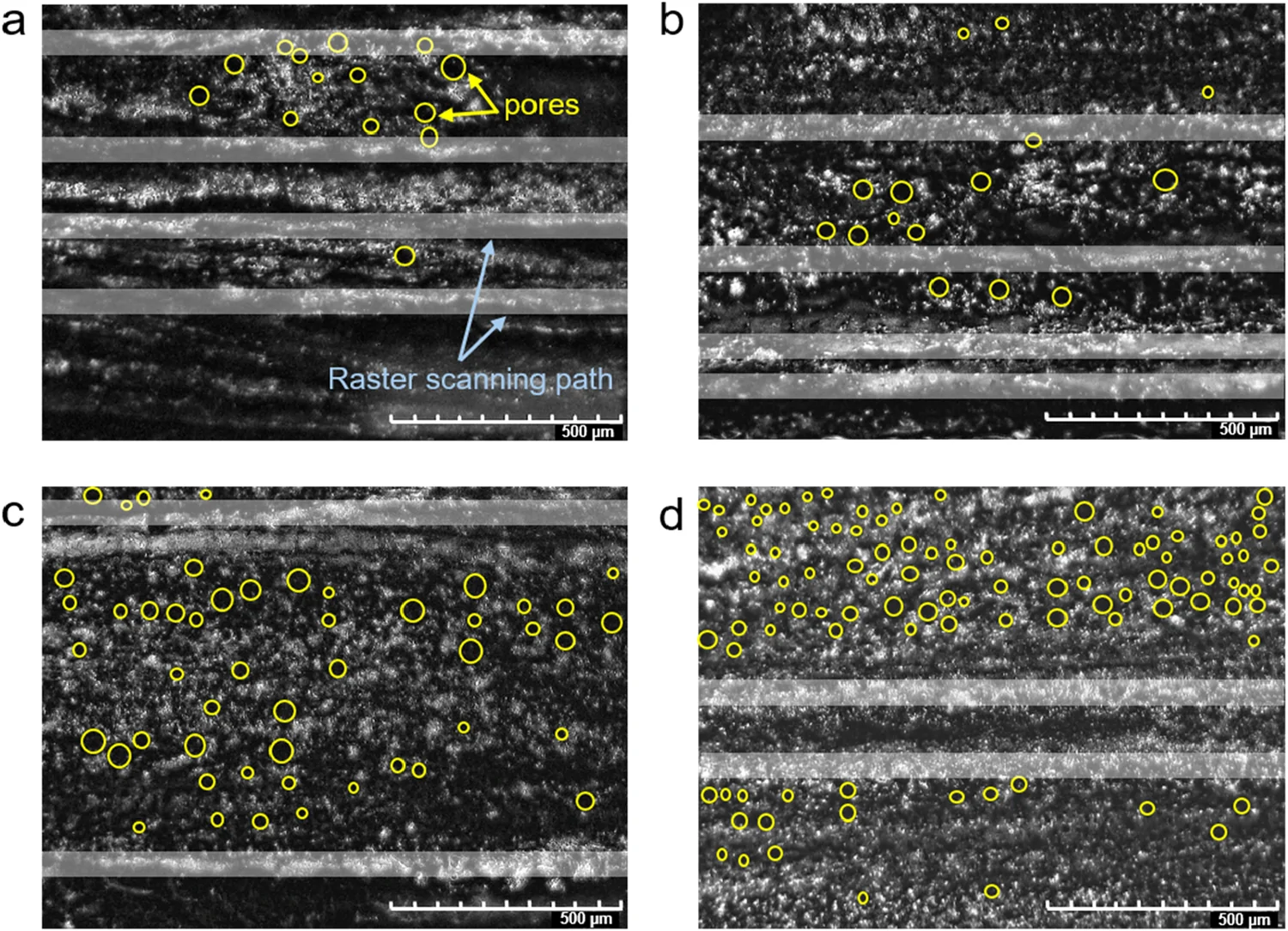
Optical microscopy images of LIG electrodes with visible pores and the raster scanning directions: (a) LIG-4.4-150, (b) LIG-5.6-150, (c) LIG-6.8-250, and (d) LIG-9.2-350. Scale bar: 500 μm.

SEM images confirmed that all LIG samples exhibited porous microstructures, consistent with the optical microscopy observations (Figure 6a–d). The pore formation can be attributed to the explosion of trapped gas pockets created by the outgassing of nitrogen and oxygen molecules during graphitization of the polyimide film.^40^ During rapid photothermal decomposition of PI by the laser energy, the polymer structure breaks down and releases volatile gaseous products, while the remaining carbon-rich material undergoes carbonization and graphitization. The rapid evolution and expansion of these gases through the carbonizing layer contribute to the formation of the porous LIG structure.^45^ Although all specimens showed porous structures, their microstructures differed substantially. To be specific, LIG-4.4-150 and LIG-5.6-150 exhibited heterogeneous microstructures with coexisting sheet-like and porous regions,^46^ indicating nonuniform laser-induced conversion across the electrode. In contrast, LIG-6.8-250 and LIG-9.2-350 showed more uniformly developed three-dimensional porous graphitic carbon networks with numerous open pores separated by thin carbon walls. Such interconnected porous structures can possibly provide a larger electrolyte-accessible surface area and facilitate electrolyte penetration into the electrode.^20^ The observed morphological differences can be attributed to variations in laser energy input, degree of carbonization, and gas evolution during laser conversion.^18,29,47^ These observations are consistent with previous studies showing that laser-processing parameters strongly affect resulting LIG morphology and porous network formation.^29,40^

**Figure 6:**
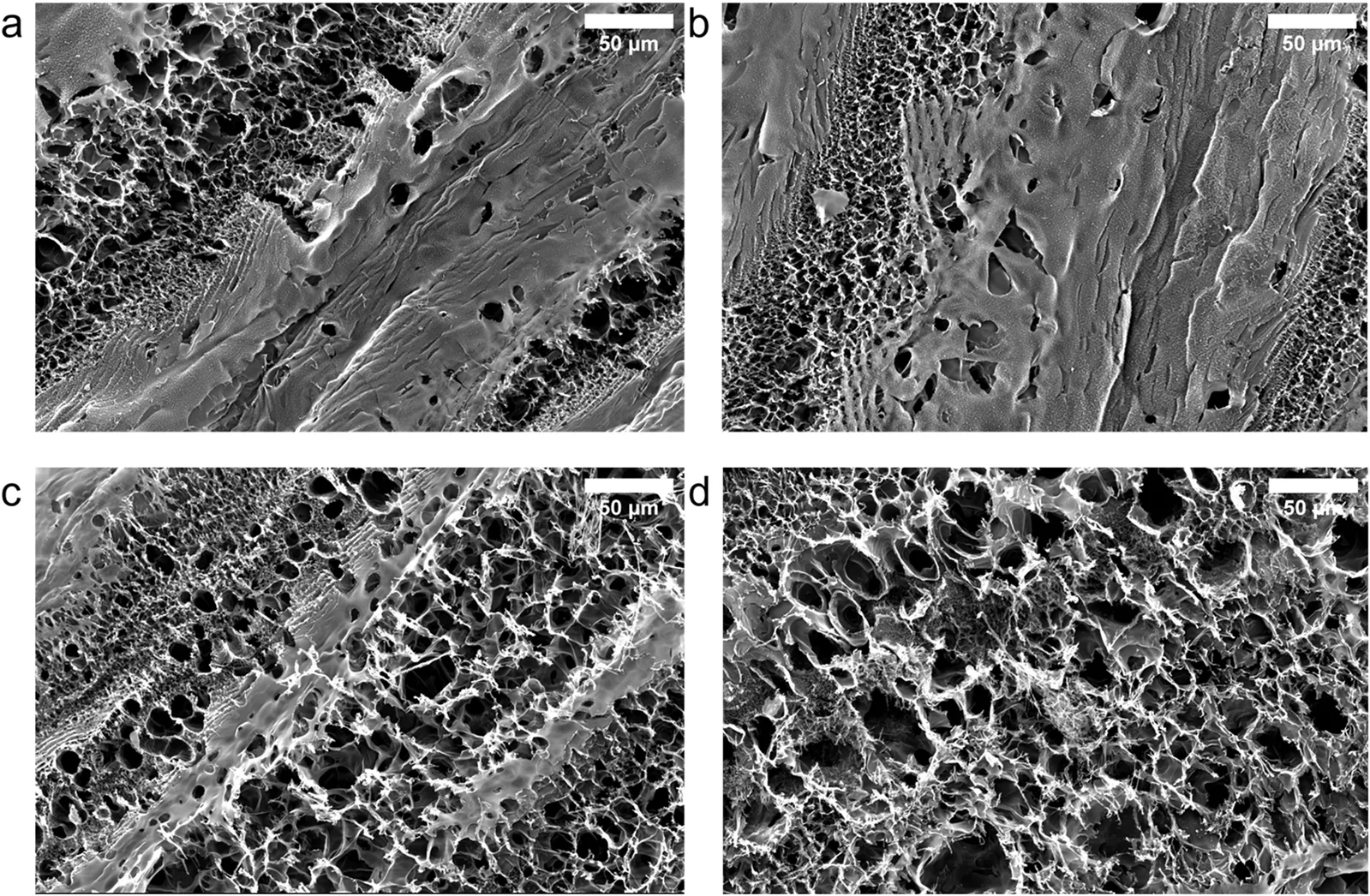
SEM images of different LIG electrodes fabricated under different laser-processing conditions: (a) LIG-4.4-150, (b) LIG-5.6-150, (c) LIG-6.8-250, (d) LIG-9.2-350. Scale bar: 50 μm.

SEM images were further analyzed using ImageJ software to estimate the apparent surface porosity of the LIG electrodes (Figure S3, Supporting Information). The SEM images were first converted to 8-bit grayscale (Figure S3e–h, Supporting Information). Pore regions were segmented from the solid LIG surface by manually adjusting the threshold to convert images into binary images until the threshold regions corresponded to pore openings observed in the original SEM images (Figure S3i–l, Supporting Information).^33^ The white areas represented the pores, while the black areas represented the solid surface of LIG. After thresholding, the identified pore area and total image area were used to calculate the apparent surface porosity

3 $$P_{\mathrm{app}}\;\left(\%\right)=\frac{\sum_{i=1}^{n}A_{pore}}{A_{total}}\times 100$$

where *A*_pore_ is the area of each selected pore, *n* is the total number of selected pore areas, and *A*_total_ is the total SEM image area. LIG-9.2-350 exhibited the highest porosity of 46.93%, whereas LIG-5.6-150 showed the lowest porosity of 18.60% (Table 1). The apparent porosities of LIG-6.8-250 and LIG-4.4-150 were 38.07% and 26.44%, respectively. These SEM observations and porosity analyses indicate that the laser-processing conditions produced LIG electrodes with distinct surface morphologies and different degrees of pore development.

**Table 1:** Apparent Surface Porosity (%) of LIG Electrodes

Samples	Porosity (%)
LIG-4.4-150	26.44
LIG-5.6-150	18.60
LIG-6.8-250	38.07
LIG-9.2-350	46.93

N_2_ adsorption–desorption measurements were performed for the LIG electrodes to determine their Brunauer–Emmett–Teller (BET) specific surface areas and pore-size distribution. SEM-derived apparent porosity represents the two-dimensional fraction of visible pore area at the imaged surface, whereas BET specific surface area reflects the surface accessible to N_2_ adsorption. The specific surface area differed noticeably among the LIG specimens, indicating the different laser-processing conditions produced measurable differences in N_2_-accessible surface area of the LIG structures (Table 2). LIG-9.2-350 showed the highest N_2_-accessible specific surface area, followed by LIG-4.4-150 and LIG-6.8-250, while LIG-5.6-150 exhibited the lowest value. This overall trend is consistent with the SEM observation; however, the BET specific surface areas do not exactly follow the SEM-derived apparent porosity percentages because these two techniques probe different structural characteristics. The relatively higher BET surface area of LIG-4.4-150 may also be partly attributed to partial detachment of some LIG samples from the underlying PI substrate after laser engraving, which could expose additional surface area to N_2_ adsorption.

**Table 2:** BET Specific Surface Areas of the LIG Electrodes

Sample	Specific surface area (m^2^/g)
LIG-4.4-150	50.90
LIG-5.6-150	42.56
LIG-6.8-250	49.94
LIG-9.2-350	75.61

Barrett–Joyner–Halenda (BJH) pore-size distributions were obtained from the adsorption branch of the N_2_ isotherms (Figure 7). Here, dV/dD represents the differential pore volume with respect to pore diameter, where higher dV/dD values indicate a greater pore-volume contribution around the corresponding pore diameter. All four LIG conditions exhibited substantial pore-volume contributions within the mesoporous range (2–50 nm). LIG-9.2-350 showed a dominant contribution at approximately 8.54 nm, while LIG-6.8-250 exhibited a broader distribution over approximately 7–21 nm. LIG-4.4-150 and LIG-5.6-150 showed dominant contributions at approximately 14.15 and 10.64 nm, respectively. These results further demonstrate that different laser-processing conditions produced distinct mesoporous characteristics in the LIG electrodes.

**Figure 7:**
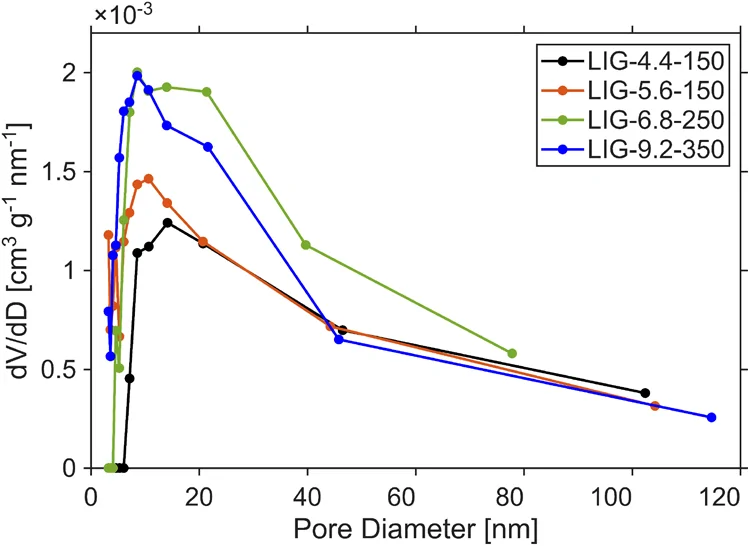
BJH pore-size distributions of the LIG-4.4-150, LIG-5.6-150, LIG-6.8-250, and LIG-9.2-350 electrodes, calculated from the adsorption branch of the N_2_ adsorption isotherms.

#### 3.3.2 Surface Morphology of PANi/LIG Electrodes

SEM images of PANi/LIG electrodes were obtained to evaluate how laser-processing-dependent LIG properties influenced PANi electrodeposition (Figure 8a–d). All samples showed nanofibrous PANi networks on the LIG surface without obvious granular aggregates, indicating that the selected electropolymerization conditions promoted controlled PANi nucleation and fiber-like growth while suppressing secondary granular overgrowth.^48,49^ However, the PANi morphology varied depending on the underlying LIG electrodes. PANi/LIG-4.4-150 (Figure 8a) showed nanofibrous PANi with localized thick clusters, and this clustered morphology became more pronounced in PANi/LIG-5.6-150 (Figure 8b). In contrast, PANi/LIG-6.8-250 (Figure 8c) exhibited a more distributed nanofibrous PANi network with less localized clusters, and PANi/LIG-9.2-350 (Figure 8d) showed the most uniform fibrous PANi coverage across the LIG surface. Since PANi was electropolymerized directly on the LIG electrode, laser-processing-dependent LIG characteristics, including electrical properties, porosity, and pore accessibility of the LIG track, can influence PANi nucleation and growth by affecting electrolyte and monomer access and local electrochemical reaction rates at the electrode surface.^50,51^ The more uniform PANi coverage observed on PANi/LIG-9.2-350 can be attributed to a more continuous and accessible porous LIG structure with relatively lower sheet resistance (Figure 8d), which can facilitate more homogeneous PANi deposition.^52^

**Figure 8:**
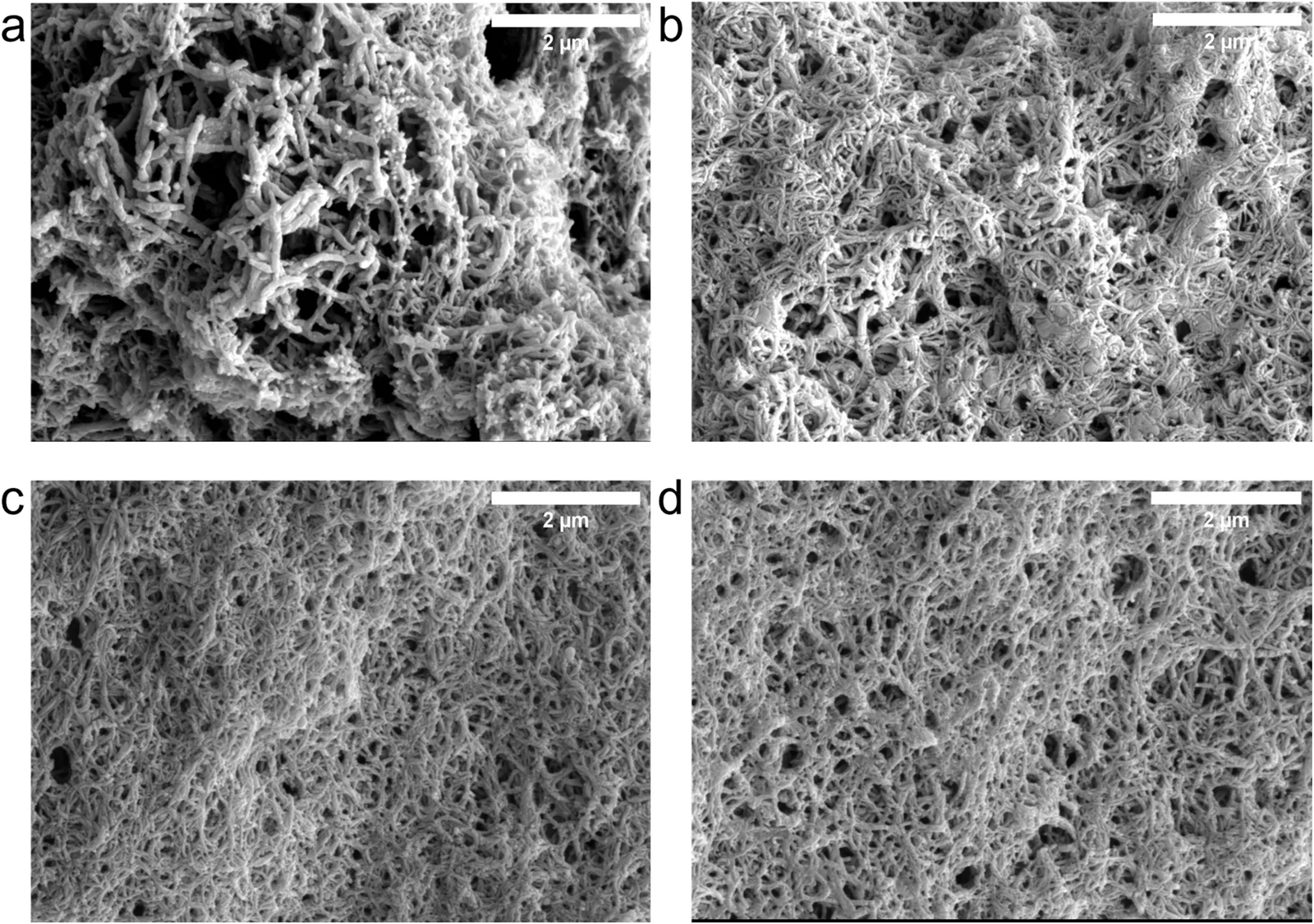
SEM images of PANi/LIG electrodes. (a) PANi/LIG-4.4-150, (b) PANi/LIG-5.6-150, (c) PANi/LIG-6.8-250, (d) PANi/LIG-9.2-350. Scale bar: 2 μm.

To further evaluate the spatial distribution of PANi on the LIG surface, EDS elemental mapping was performed (Figure S4, Supporting Information). The EDS nitrogen (N) maps showed clear differences in the spatial distribution of nitrogen-rich PANi regions among the PANi/LIG electrodes (Figure 9e–h). Since N is associated with PANi, a more uniform N distribution indicates more uniform PANi deposition on the LIG surface. To quantitatively compare PANi distribution, the N elemental maps were processed using image analysis (Figure 9e–h).^53^ The RGB images of EDS N maps were first converted into binary images, in which white pixels indicate N-rich regions, and black pixels represent the surrounding matrix (Figure 9i–l). The binary images were then divided into 8 × 5 equal units, and the number of N pixels in each unit was calculated. The standard deviation (SD) of the N-pixel counts was calculated using eq 4

4 $$SD=\sqrt{\frac{1}{n-1}\sum_{i=1}^{n}{\left(N_{i}-N_{M}\right)}^{2}}$$

where *n* is the number of units (*n* = 40), *N*_*i*_ is the number of nitrogen pixels in the *i*-th unit (*i* = 1, 2, ..., 40), and *N*_M_ is the mean number of nitrogen pixels across the 40 units. Because the total *N* signal may vary among EDS maps, the coefficient of variation (COV) was used to compare the relative uniformity of *N* distribution using eq 5

5 $$\mathrm{CO}V=\frac{S_{D}}{N_{M}}$$

**Figure 9:**
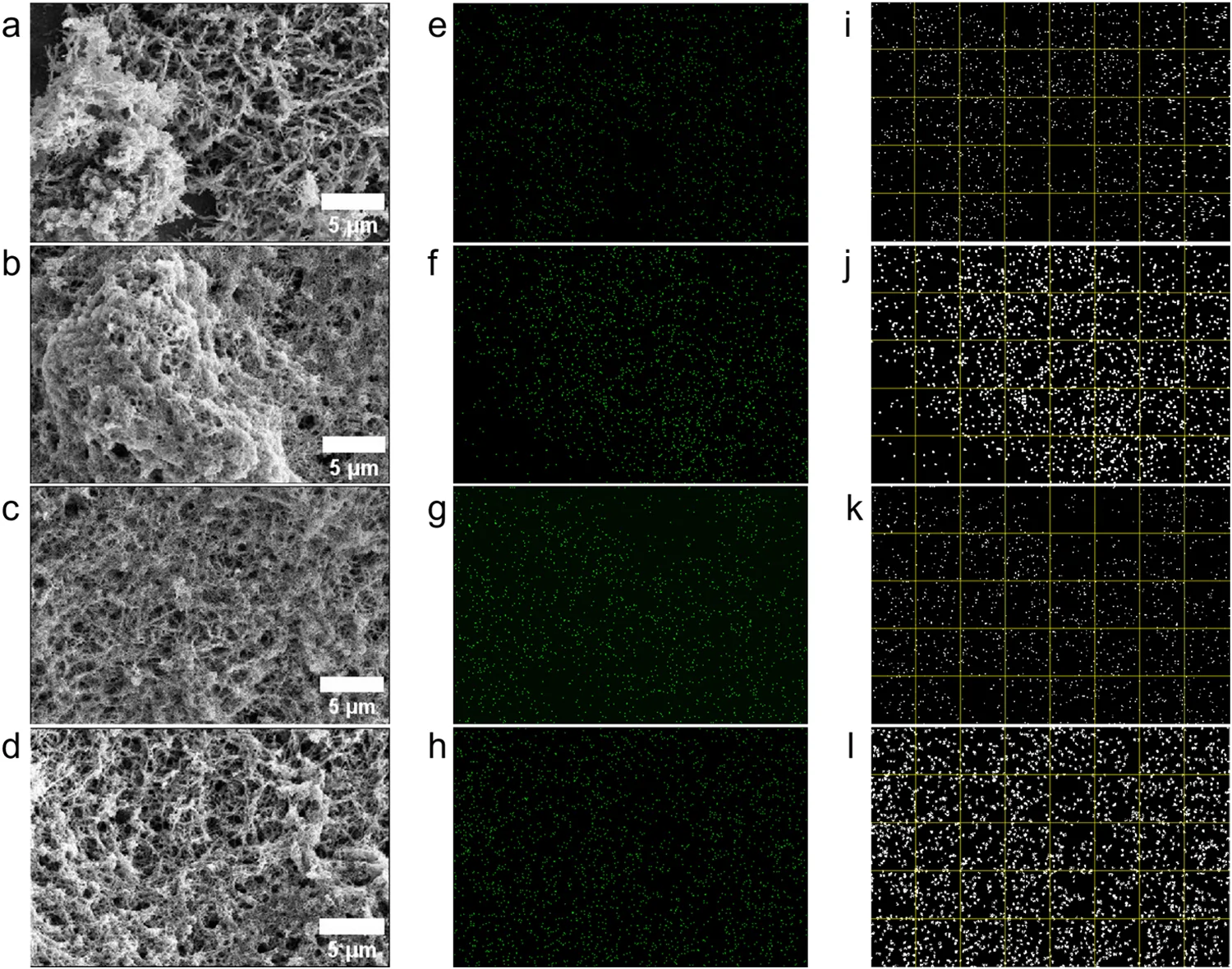
SEM images, EDS N elemental maps, and binary N-map analysis of PANi/LIG electrodes. SEM images of (a) PANi/LIG-4.4-150, (b) PANi/LIG-5.6-150, (c) PANi/LIG-6.8-250, and (d) PANi/LIG-9.2-350; (e–h) corresponding N elemental maps; and (i–l) binary N maps divided into 8 × 5 grids for coefficient of variation (COV) analysis.

A lower magnitude of COV indicates more uniform deposition of N elements, whereas a higher COV indicates greater nitrogen clustering.^53,54^ PANi/LIG-9.2-350 showed the lowest COV value, and highest COV value was found for PANi/LIG-5.6-150 (Table 3). The results agree with the SEM images of PANi/LIG (Figure 8a–d), where PANi/LIG-9.2-350 has the most uniform deposition of PANi molecules, while PANi/LIG-5.6-150 has the most pronounced clustered PANi deposition.

**Table 3:** Coefficient of Variation (COV) of the N-Elemental Maps for PANi/LIG Electrodes

Samples	Coefficient of Variation (COV) of N map
PANi/LIG-4.4-150	0.39
PANi/LIG-5.6-150	0.414
PANi/LIG-6.8-250	0.311
PANi/LIG-9.2-350	0.171

To quantify the surface pore-size distributions of the PANi/LIG electrodes, pore openings observed in the SEM images were analyzed using ImageJ. The measured pore areas were converted to equivalent circular diameters, and histograms of pore number as a function of equivalent pore diameter were generated.

All PANi/LIG electrodes exhibited right-skewed pore-size distributions dominated by smaller surface pore openings (<100 μm) with progressively fewer pores at larger equivalent diameters (Figure 10a–d). However, the distributions differed among the four electrode conditions. PANi/LIG-5.6-150 and PANi/LIG-6.8-250 showed pore populations concentrated mainly at smaller diameters. PANi/LIG-4.4-150 had the lowest overall number (*n* = 505) of detected surface pores but the broadest size range extending beyond 400 μm. PANi/LIG-9.2-350 exhibited the highest number of detected surface pores (*n* = 1,703) among the four electrodes, with most pores occurring below approximately 150 μm. These results indicate that the different LIG processing conditions produced PANi/LIG surfaces with distinct pore-size characteristics after PANi electrodeposition.

**Figure 10:**
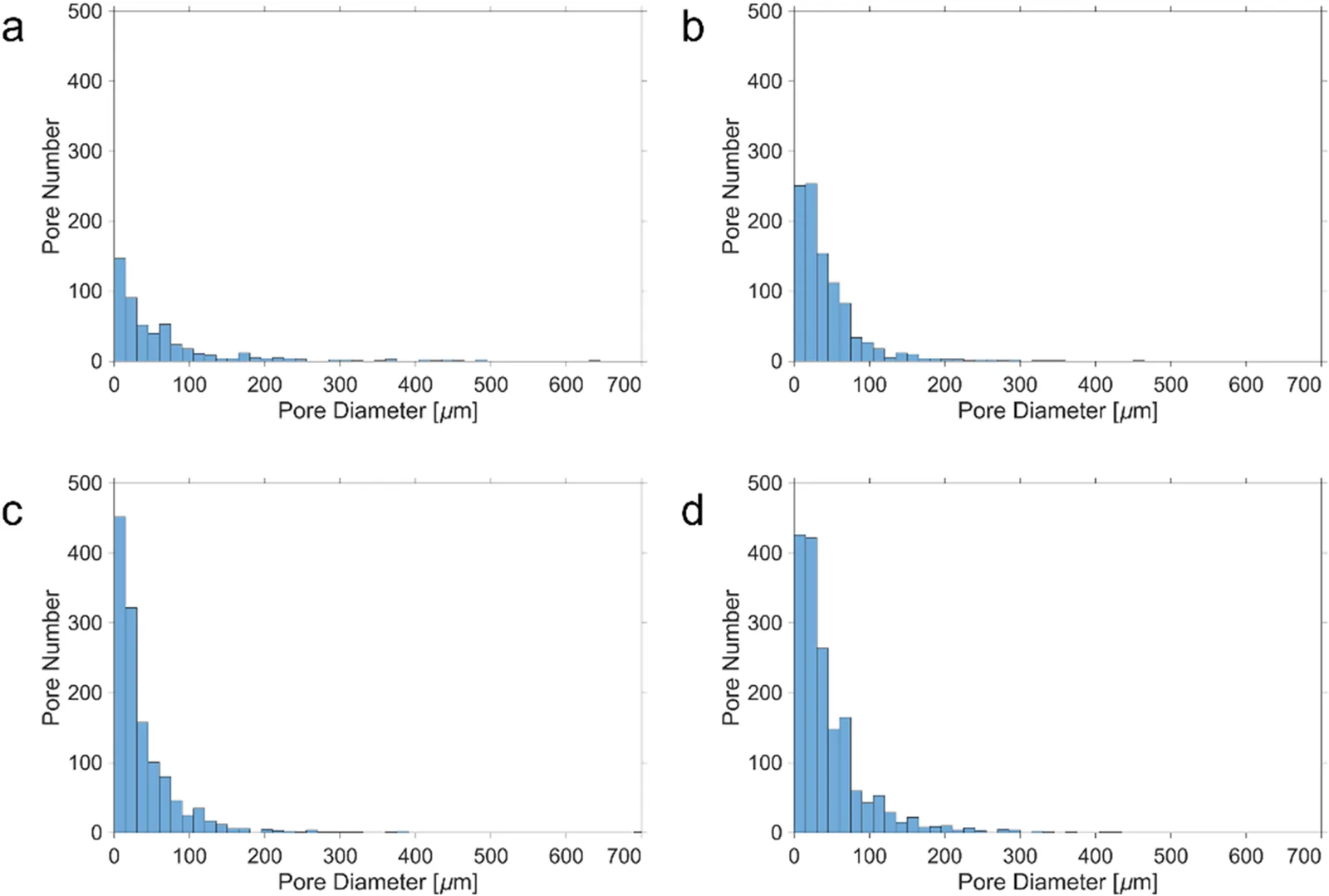
Pore-size distributions of PANi/LIG electrode surfaces obtained from SEM image analysis. (a) PANi/LIG-4.4-150, (b) PANi/LIG-5.6-150, (c) PANi/LIG-6.8-250, and (d) PANi/LIG-9.2-350.

#### 3.3.3 Surface-Chemical Characterization of LIG and PANi/LIG Electrodes

XPS survey spectra were obtained from representative LIG-4.4-150 and PANi/LIG-4.4-150 to confirm PANi deposition on the LIG surface (Figure 11). In the LIG-4.4-150 spectrum, the dominant C 1s and O 1s peaks at 285 and 531 eV, respectively, indicate a carbon-rich LIG surface with detectable oxygen-containing species.^55^ After electropolymerization, the PANi/LIG-4.4-150 showed a clear N 1s peak at ∼400 eV, coming from the PANi after electropolymerization.^56^ The PANi/LIG-4.4-150 electrode exhibited an N/C ratio of 0.15:1, which is close to the theoretical N/C ratio (0.167:1) of pure PANi.^57^ Considering that XPS probes only near-surface region, with a typical information depth of less than 8 nm, this indicates that the XPS-detected surface was dominated by a PANi-rich layer after electropolymerization, further supporting successful PANi deposition on the LIG surface.

**Figure 11:**
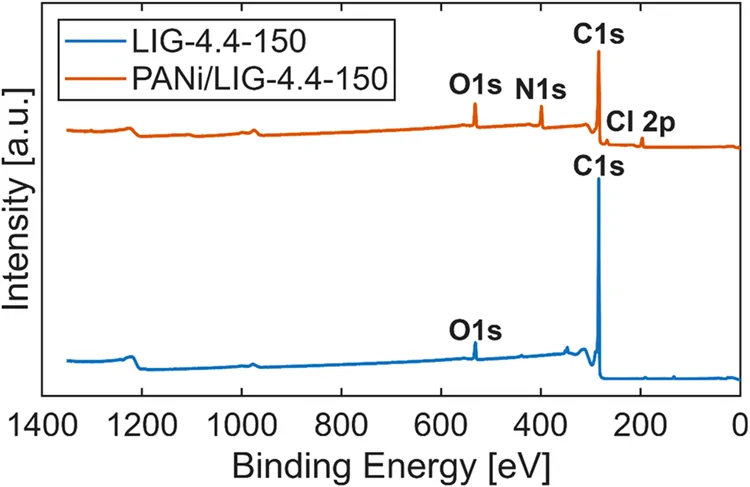
XPS survey spectra of LIG-4.4-150 and PANi/LIG-4.4-150, showing C 1s and O 1s peaks in both samples and additional N 1s and Cl 2p peaks in PANi/LIG after PANi electrodeposition.

#### 3.3.4 Structural Characterization of LIG and PANi/LIG Electrodes

Raman spectroscopy was performed to investigate the structural characteristics of the LIG electrodes. All LIG samples exhibited characteristic D (∼1350 cm^–1^), G (∼1580 cm^–1^), and 2D (∼2700 cm^–1^) bands, indicating the formation of sp^2^-rich graphitic carbon, consistent with previous studies (Figure 12).^45,58^ The D, G, and 2D bands correspond to defect-activated disordered sp2 carbon, in-plane stretching of sp2 carbon, and a second-order two-phonon resonance sensitive to graphene layer number and stacking, respectively.^59,60^ The *I*(D)/*I*(G) ratios ranged from 0.34 to 0.77, showing different degrees of structural disorder among the samples. LIG-9.2-350 exhibited the lowest *I*(D)/*I*(G) ratio of 0.34 and a relatively narrow D-band fwhm, indicating lower structural disorder and a more consistent sp2 carbon network. In contrast, LIG-6.8-250, LIG-5.6-150, and LIG-4.4-150 showed higher I­(D)/I­(G) ratios of 0.70, 0.73, and 0.77, respectively, indicating higher defect density.

**Figure 12:**
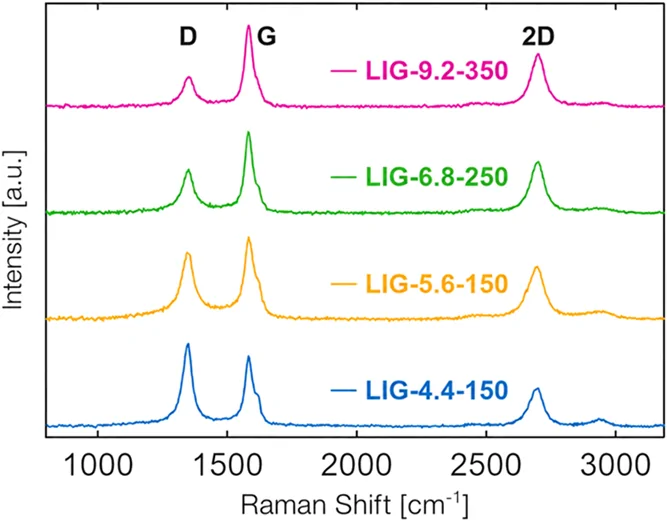
Raman spectra of LIG samples fabricated under different laser-processing conditions.

LIG-6.8–250 showed the highest *I*(2D)/*I*(G) ratio of 0.92 and the narrowest G-band fwhm of 27.1 cm^–1^, suggesting more developed graphitic domains.^61^ However, its sheet resistance was higher than that of LIG-9.2-350 and LIG-4.4-150, likely because discontinuities in the LIG track interrupted conductive pathways (Figure 13).^18^ Similarly, although LIG-9.2-350 had a lower *I*(2D)/*I*(G) ratio of 0.66 than LIG-6.8-250, its continuous and uniformly porous carbon network provided interconnected conductive pathways, resulting in low sheet resistance. These results indicate that the sheet resistance of the LIG electrodes was influenced not only by graphitic ordering but also by the continuity and connectivity of the carbon network of LIG track.

**Figure 13:**
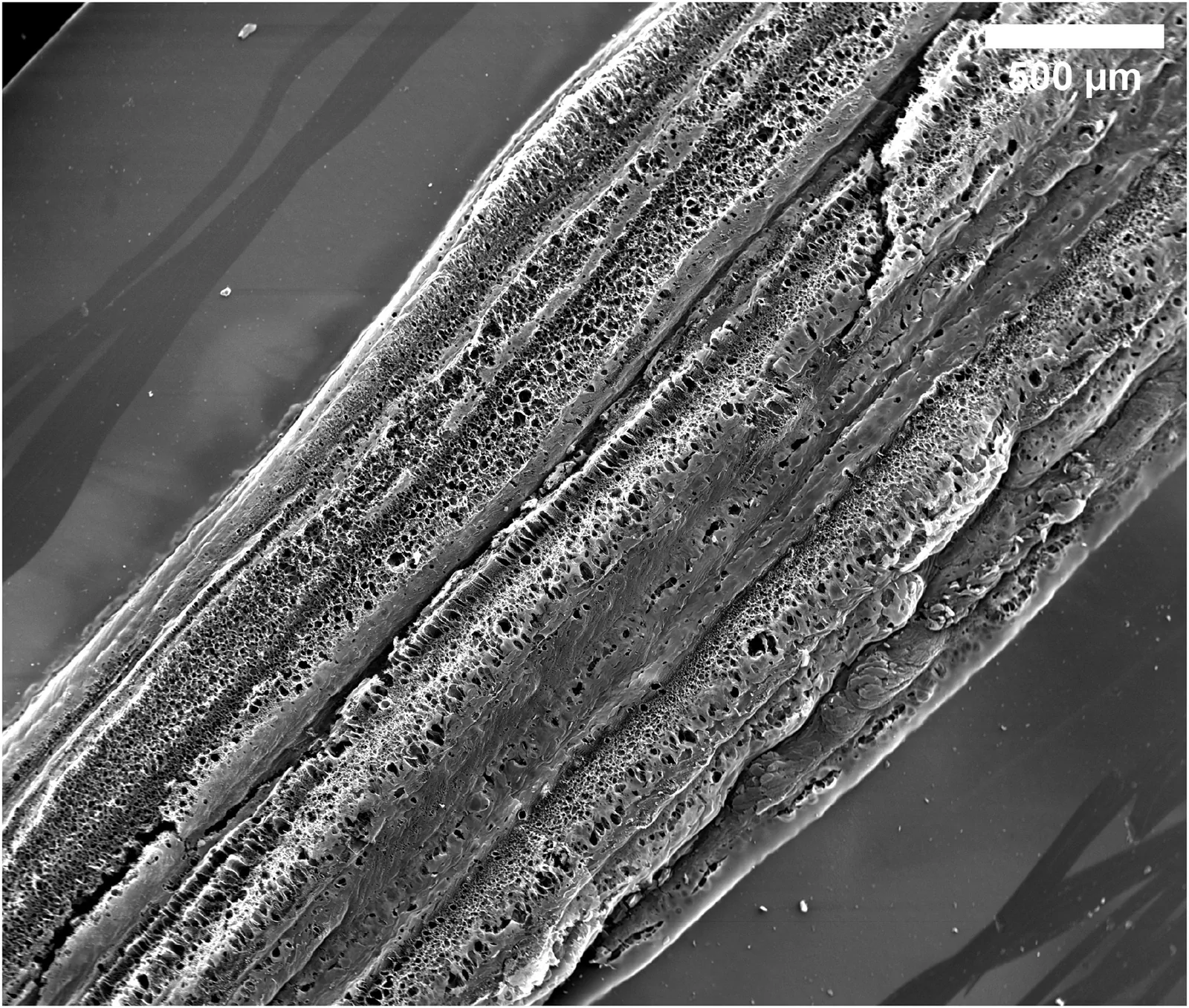
SEM image of LIG-6.8-250 at lower magnification (×30) showing discontinuities in the LIG track. Scale bar: 500 μm.

The Raman spectra of PANi/LIG electrodes are shown in Figure 14. All PANi/LIG electrodes exhibited characteristic PANi vibrational bands between 1100 and 1650 cm^–1^, further confirming PANi formation on the LIG surface.^55^ PANi/LIG-9.2-350 exhibited a 2D band near 2700 cm^–1^ that was not clearly observed in the other PANi/LIG electrodes. This band is attributed to the 2D band of the underlying LIG, suggesting that the PANi layer on LIG-9.2-350 may be relatively open, porous, or thinner in some regions, allowing Raman detection of the underlying LIG. This interpretation aligns with the SEM-observed porous PANi/LIG morphology.

**Figure 14:**
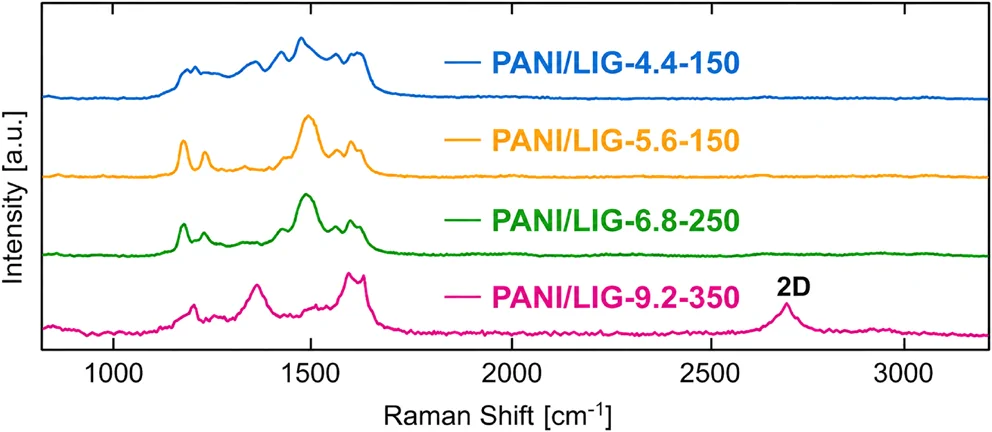
Raman spectra of PANi on LIG substrates fabricated under different laser-processing conditions.

The Raman spectra of the PANi/LIG electrodes were deconvoluted using Fityk with linear baseline correction and Gaussian peak fitting (Figure 15a–d). The peak at ∼1160 cm^–1^ is assigned to C–H bending of the benzene ring, while the band at 1215 cm^–1^ corresponds to C–N stretching. The bands at ∼1330 and ∼1420 cm^–1^ are associated with C–N^+^ stretching and C–N stretching in polaronic structures, respectively, indicating the presence of polaronic PANi. The bands at ∼1480, ∼1550, and ∼1600 cm^–1^ are assigned to C=N stretching, C–C stretching, and C=C stretching vibrations of quinoid/benzenoid rings, respectively.^62,63^ It should be noted that the graphitic D and G bands of the underlying LIG, located near 1350 and 1580 cm^–1^, respectively, can overlap with the C–N^+^ polaronic band near 1330 cm^–1^ and quinoid/benzenoid ring vibrations near 1600 cm^–1^.^64^ The deconvoluted Raman spectra showed that the relative peak intensities of the characteristic PANi bands varied among the PANi/LIG electrodes. Variations in the C–N stretching, C–N^+^ stretching, quinoid C = N stretching, and polaronic C–N peaks can reflect differences in the relative contributions of the EB and ES states, oxidation states, and polaronic character of the PANi layers.^65,66^ To provide a quantitative comparison of the polaron-associated Raman contribution, the fitted peak areas of the two polaron-related bands at 1338 cm^–1^ and 1406 cm^–1^ were summed and normalized to the total fitted Raman peak area

6 $$\mathrm{Spectral weight}=\frac{\mathrm{Peak area}}{\mathrm{Total area of all fitted peaks}}\times 100$$

**Figure 15:**
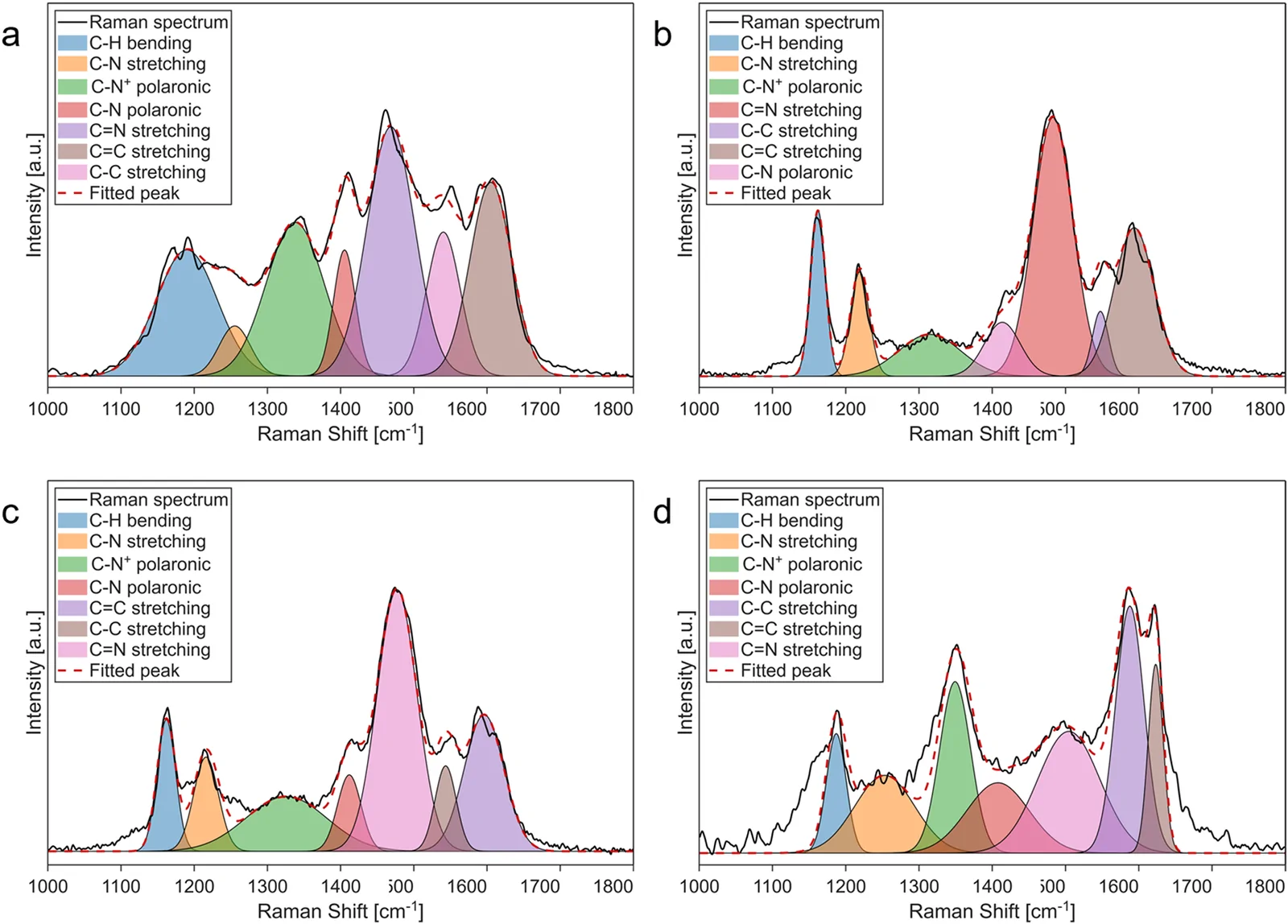
Raman peak deconvolution of PANi/LIG samples fabricated using different LIG processing conditions: (a) PANi/LIG-4.4-150, (b) PANi/LIG-5.6-150, (c) PANi/LIG-6.8-250, and (d) PANi/LIG-9.2-350.

The resulting relative polaron-associated spectral weights were 25.51%, 17.30%, 21.74%, and 28.40% for PANi/LIG-4.4-150, PANi/LIG-5.6-150, PANi/LIG-6.8-250, and PANi/LIG-9.2-350, respectively, with PANi/LIG-9.2-350 exhibiting the highest relative polaronic contribution (Table S2, Supporting Information). The differences support variations in the relative polaronic character of PANi among the four electrodes.^38^ Because PANi-related bands could overlap with the underlying LIG D and G bands, particularly near 1350 and 1580 cm^–1^, these values should be interpreted as relative Raman spectral contributions rather than absolute measures of PANi protonation degree.

To compare the relative phenylenediamine and quinonimine-related contributions in PANi, the deconvoluted C–N and C=N Raman peak areas were analyzed. These band assignments are supported by previous vibrational studies, which have assigned the C–N stretching band at approximately 1220–1260 cm^–1^ to reduced amine/benzenoid structures and the C=N stretching band at approximately 1470–1500 cm^–1^ to oxidized quinonimine structures.^63^ The resulting C–N/C=N peak-area ratios were 0.133, 0.189, 0.225, and 0.613 for PANi/LIG-4.4-150, PANi/LIG-5.6-150, PANi/LIG-6.8-250, and PANi/LIG-9.2-350, respectively, indicating differences in the relative redox structural characteristics of PANi deposited on LIG substrates.

Raman Spectroscopy was further evaluated to compare the relative polaron- and bipolaron-associated Raman contributions in PANi by analyzing the deconvoluted Raman peak areas at approximately 1406 and 1468 cm^–1^, respectively. The resulting polaron/bipolaron peak-area ratios were 1.83, 1.35, 0.43, and 0.70 for PANi/LIG-4.4-150, PANi/LIG-5.6-150, PANi/LIG-6.8-250, and PANi/LIG-9.2-350, respectively, indicating differences in the relative charged-state contributions of PANi deposited on the four LIG conditions.

### 3.4 pH-Sensing Performance of PANi/LIG pH Sensors

The potentiometric responses of all PANi/LIG sensors showed linearly decreasing EMF values as pH increased from 4 to 9 (*R* ^2^ ≥ 0.997; Figure 16a–d), demonstrating their ability to transduce changes in proton activity into measurable electrical potential signals. Each data point represents the mean EMF value obtained from three sensors fabricated under the identical laser-processing conditions, and the error bars indicate the standard error of the mean. Representative time-dependent EMF responses recorded over the pH 4–9 range are provided in Figure S5a–d (Supporting Information). The pH-sensing response is attributed to the reversible protonation and deprotonation of PANi, which modulates the interfacial potential of the PANi/LIG sensing layer relative to the Ag/AgCl reference electrode (Figure 17). As pH increases and proton activity decreases, PANi becomes progressively deprotonated and shifts toward the emeraldine base (EB) state, resulting in lower EMF values.^47,67^ The pH sensitivity was estimated from the slope of the linear regression line fitted to the data. Although identical PANi electropolymerization conditions were used, the PANi-modified LIG sensors exhibited different pH sensitivities depending on the LIG electrode used as the transducing layer. LIG-4.4-150 and LIG-5.6-150 exhibited sub-Nernstian sensitivities of −52.38 mV/pH and −45.22 mV/pH, respectively, compared with the theoretical Nernstian response of −59.2 mV/pH at 25 *°C*. In contrast, the LIG-6.8-250 sensor exhibited an improved sensitivity of −54.4 mV/pH, which is closer to the Nernstian response. The highest sensitivity was achieved by the LIG-9.2-350 sensor, which showed a near-Nernstian sensitivity of −60.04 mV/pH (Figure 16d). The enhanced sensitivity of PANi/LIG-9.2-350 can be attributed to uniformly distributed fibrous PANi morphology supported by a three-dimensional, highly porous, and interconnected LIG network (Section 3.3), which likely increased electrolyte-accessible PANi sites and promoted potentiometric transduction.^68,69^ To evaluate device-to-device reproducibility, calibration curves were also fitted independently for each sensor, and the resulting mean sensitivity and standard deviation are reported in Table S3­( Supporting Information).

**Figure 16:**
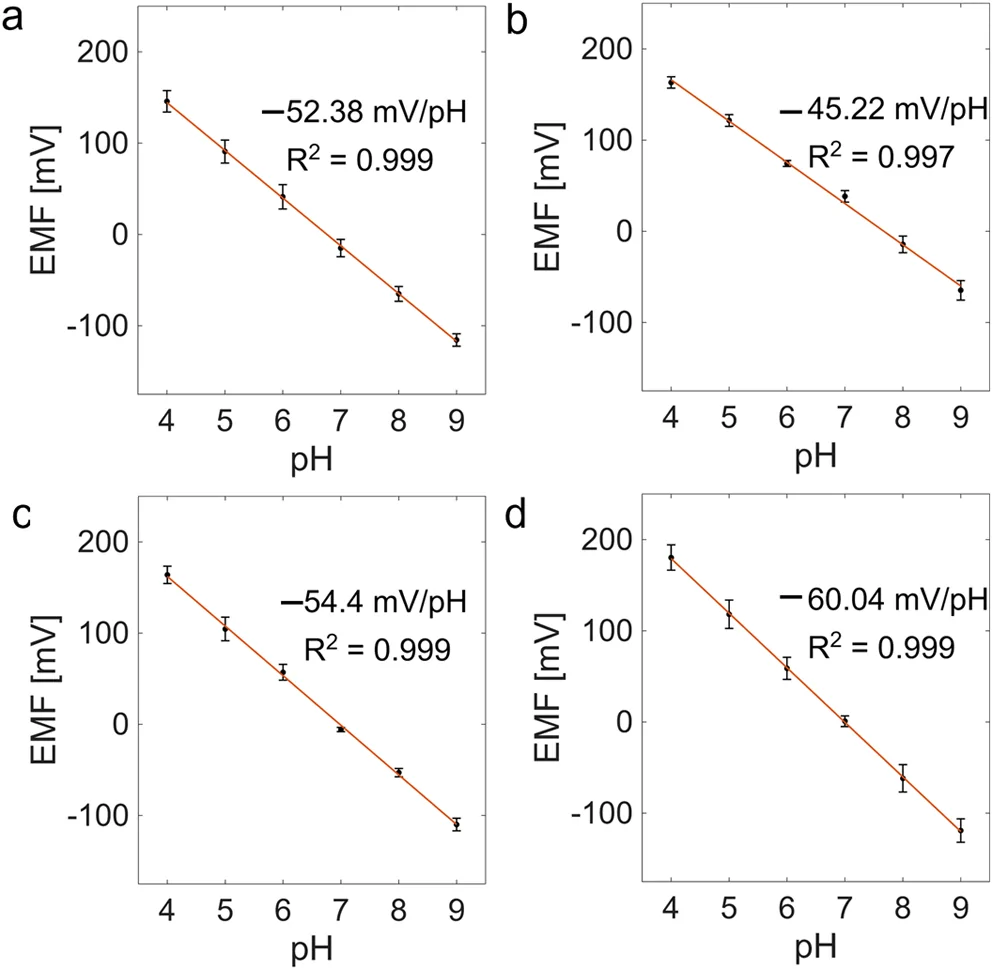
Calibration curves of EMF versus pH for PANi/LIG sensors: (a) LIG-4.4-150, (b) LIG-5.6-150, (c) LIG-6.8-250, and (d) LIG-9.2-350 (*n* = 3).

**Figure 17:**
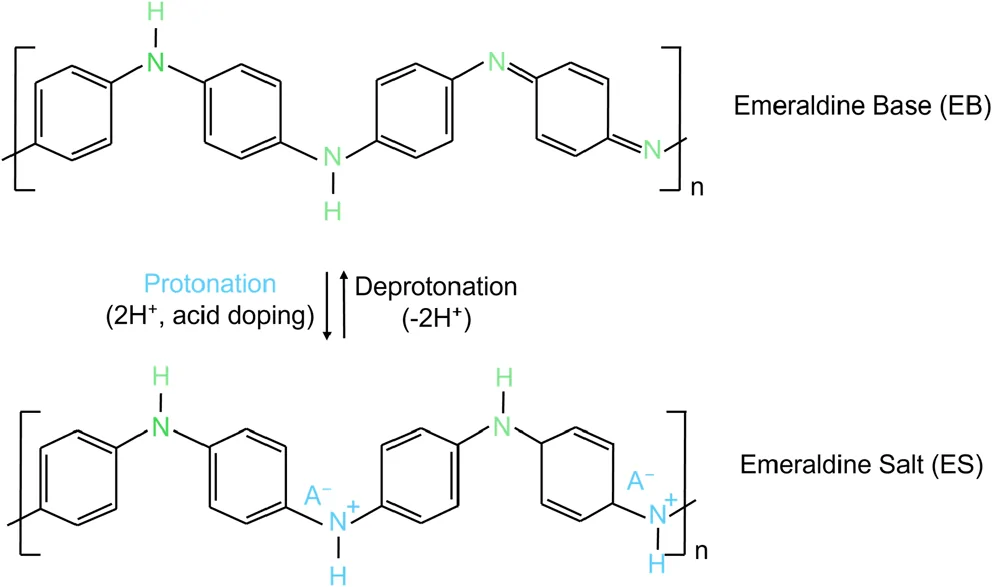
Schematic of the pH-sensing mechanism based on the reversible protonation and deprotonation of PANi between the emeraldine base (EB) and emeraldine salt (ES) states.

The 12 h drift stability of the PANi/LIG sensors was evaluated by continuously recording the EMF values in pH 7 buffer for 12 h. All sensors showed great stability, with drift rates below 3 mV/h, lower than values reported in previous studies (Figure 18a–d).^70^ PANi/LIG-6.8-250 and PANi/LIG-9.2-350 exhibited comparatively better 12 h drift stability, with drift rates of 1.50 and 1.67 mV/h, respectively. In contrast, LIG-4.4-150 and LIG-5.6-150 showed higher drift rates of 2.50 and 2.83 mV/h, respectively, indicating relatively lower baseline stability during prolonged measurements. The improved 12 h drift stability of PANi/LIG-6.8-250 and PANi/LIG-9.2-350 may be associated with their more uniform PANi coverage without thick PANi clusters (Figure 8c,d), which likely promoted a more stable PANi/electrolyte interfacial equilibrium and reduced time-dependent potential drift during 12 h measurements.^9^ It should be noted that the measured EMF drift may include contributions from both the PANi/LIG working electrode and the integrated reference electrode. However, in the integrated reference electrode, PVB-KCl layer serves as a solid-state electrolyte reservoir and diffusion layer that helps maintain a high and relatively constant chloride concentration near the Ag/AgCl interface, thereby supporting reference-potential stability.^26^

**Figure 18:**
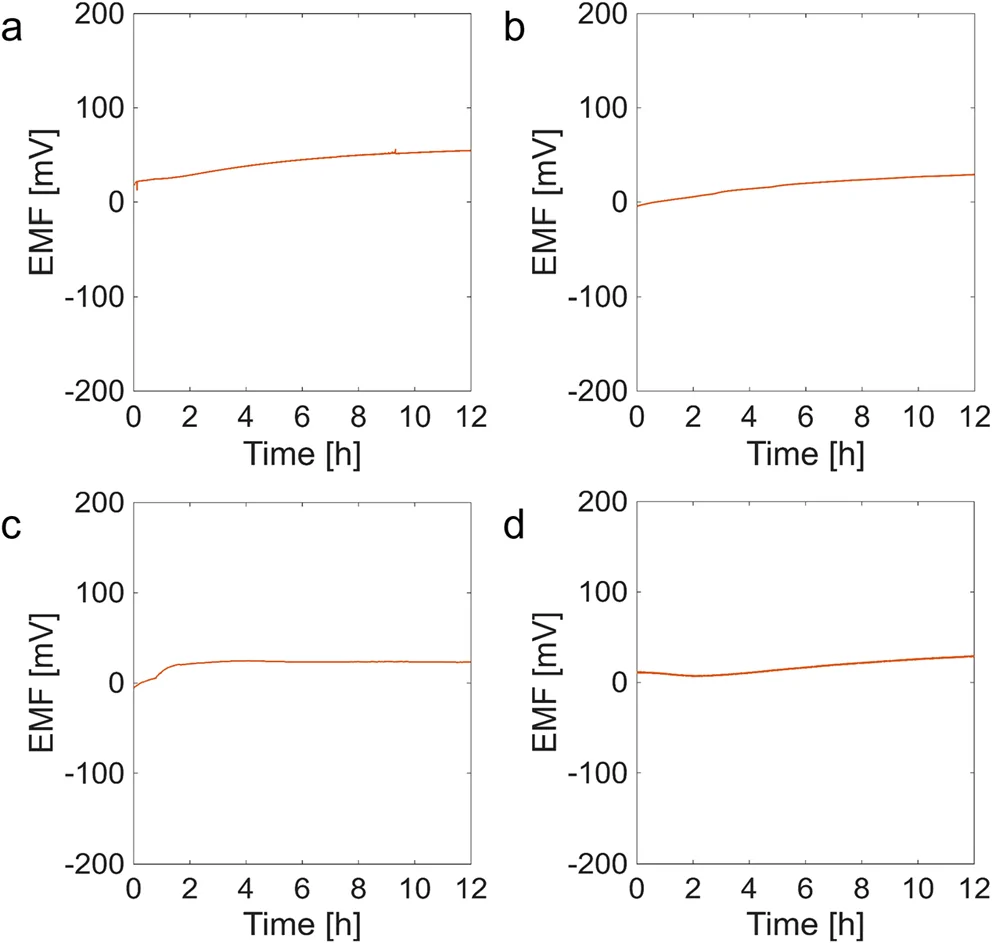
12 h drift stability of PANi/LIG sensors in pH 7 buffer solution: (a) LIG-4.4-150, (b) LIG-5.6-150, (c) LIG-6.8-250, and (d) LIG-9.2-350.

The reversibility of the PANi/LIG sensors was evaluated by dynamically cycling the sensors between pH 5, 7, and 9, as shown in Figure 19a–d. Hysteresis was calculated as the absolute EMF difference (Δ*E*) measured at the identical pH between the forward and reverse cycles (Figure 19a).^9,71^ The average hysteresis values at pH 5 and pH 7 were used to compare reversibility among the sensors. All sensors exhibited relatively low hysteresis, indicating good reversibility of the potentiometric response. PANi/LIG-9.2-350 sensor exhibited the lowest hysteresis of 2.06 mV, while the other sensors showed average hysteresis values of 3.50–3.66 mV. These hysteresis values are comparable to or lower than previously reported values.^72^ The reduced hysteresis of PANi/LIG-9.2-350 indicates more reversible protonation/deprotonation. Its more open PANi morphology and reduced localized clustering may facilitate ion transport, whereas the thicker PANi clusters in PANi/LIG-4.4-150 and PANi/LIG-5.6-150 may delay proton transport or promote partial ion trapping, thereby contributing to differences in EMF hysteresis.^73^ Future work will use frequency-dependent EIS and diffusion models to quantitatively investigate the relationship between proton-transport behavior and EMF hysteresis.

**Figure 19:**
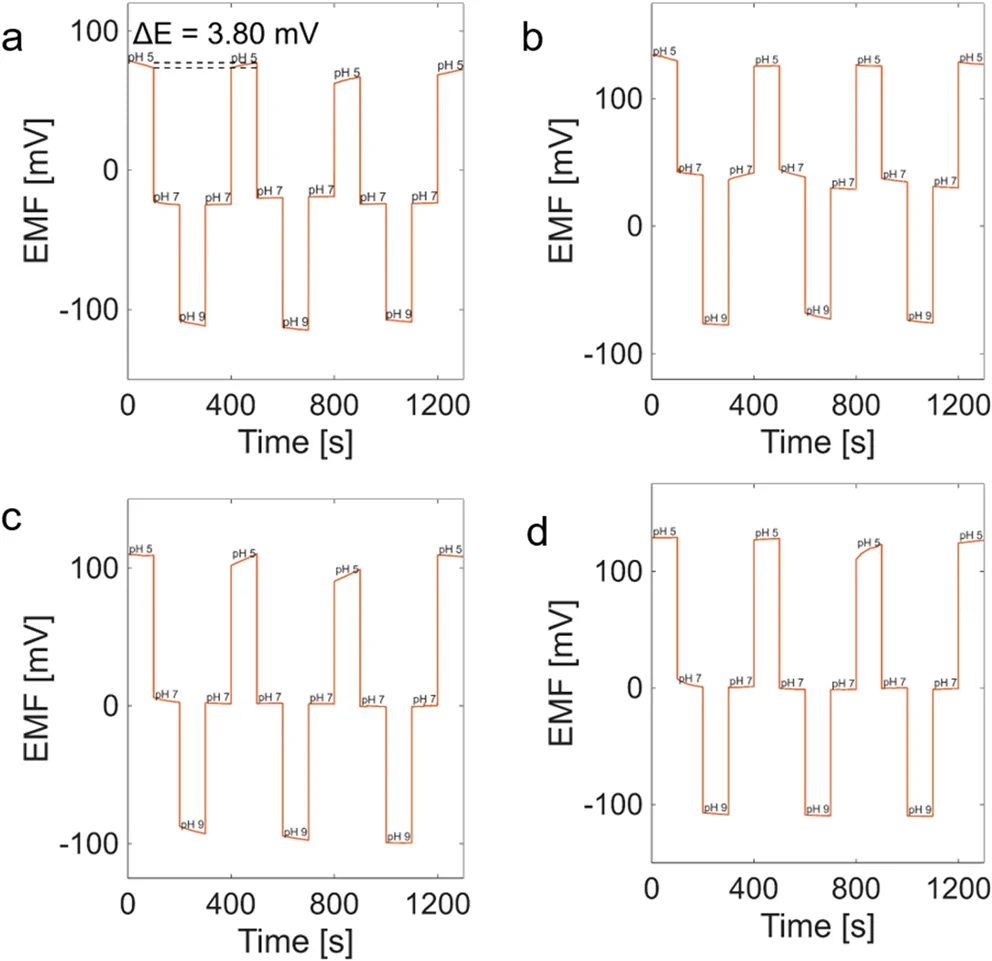
Reversibility of PANi/LIG pH sensors during cycling among pH 5, 7, and 9 buffer solutions: (a) LIG-4.4-150, (b) LIG-5.6-150, (c) LIG-6.8-250, and (d) LIG-9.2-350.

Repeatability describes the variation in sensor potential when the same pH conditions are revisited during repeated measurement cycles. Repeatability was assessed by calculating the relative standard deviation (RSD) of the EMF values recorded at pH 5 and pH 9 during the reversibility sequence and then averaging the two RSD values for each sensor (Figure 19a–d).

7 $$RSD\;\left(\%\right)=\frac{SD\;of\;EMF\;at\;each\;pH}{\left|mean\;EMF\;at\;each\;pH\right|}$$

The average RSD values were 4.35%, 4.02%, 3.87%, and 1.42% for PANi/LIG-4.4-150, PANi/LIG-5.6-150, PANi/LIG-6.8-250, and PANi/LIG-9.2-350, respectively. The lowest RSD value of PANi/LIG-9.2-350 indicated the most repeatable EMF response among the four sensors. This result is consistent with its lower hysteresis and suggests a more consistent potential response upon repeated exposure to the same pH conditions.

The response time (*t*_90_) was evaluated by immersing each sensor in pH 8 buffer under magnetic stirring at 300 rpm and then dynamically shifting the solution to pH 7 by adding 0.01 M HCl. The EMF response was continuously recorded, and t90 was defined as the time elapsed from the imposed pH change until the EMF reached 90% of the total potential change between the initial and final steady-state values, as shown in Figure 20. Among all fabricated sensors, PANi/LIG-9.2-350 exhibited the fastest response time of 13.2 s, while PANi/LIG-6.8-250, PANi/LIG-4.4-150, and PANi/LIG-5.6-150 sensors showed response times of 31, 46, and 53.6 s, respectively. The faster response time of PANi/LIG-9.2-350 may be attributed to its open porous structure, uniformly distributed nanofibrous PANi, and highly conductive LIG network.^15^ These features likely facilitated electrolyte penetration, increased proton-accessible PANi sites, shortened ion diffusion pathways, and supported faster pH-dependent EMF response. In contrast, the relatively slower responses of PANi/LIG-4.4-150 and PANi/LIG-5.6-150 may be due to lower pore accessibility and more localized PANi clustering, which can restrict electrolyte diffusion and delay protonation/deprotonation within the PANi layer.^60^

**Figure 20:**
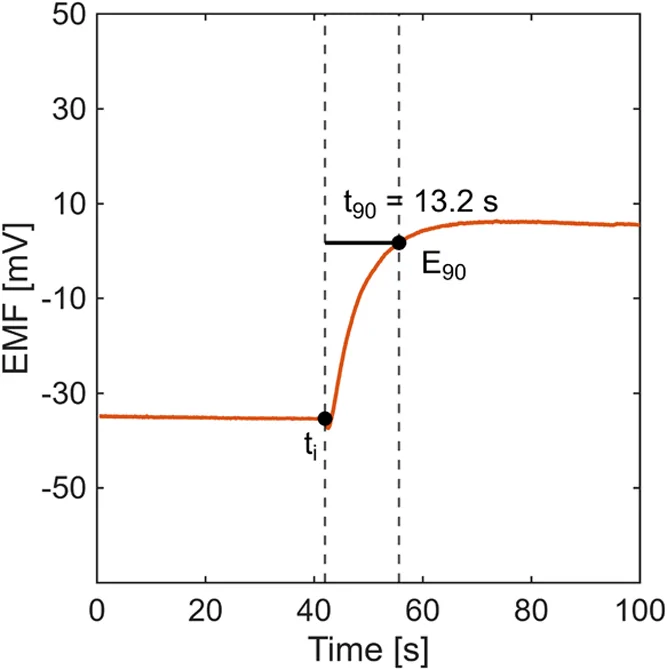
Temporal EMF response of a PANi/LIG-9.2-350 pH sensor during titration from pH 8 to pH 7.

Table 4 summarizes the pH-sensing performance of the PANi/LIG sensors. Overall, PANi/LIG-9.2-350 exhibited the best combined performance, including near-Nernstian sensitivity, low drift, low hysteresis, and fast response. Among the four laser-processing conditions, LIG-9.2–350 exhibited the highest sensitivity (−60.04 mV/pH), fastest response time (13.2 s), lowest hysteresis (2.06 mV), and relatively low drift rate (1.67 mV/h). The improved pH-sensing performance of PANi/LIG-9.2-350 can be associated with its continuous porous LIG network, low sheet resistance, and uniform fibrous PANi coverage without clustering, which together enhance electrolyte-accessible PANi sites, facilitate reversible proton exchange, and support stable EMF generation. In contrast, LIG-4.4-150 exhibited low sheet resistance (3.44 Ω/sq), relatively low apparent porosity (26.44%), and a more heterogeneous, sheet-like structure. The deposited PANi showed nanofibrous features with localized thick clusters. Although it exhibited a low sheet resistance of 3.44 Ω/sq, its relatively low apparent porosity and less uniform PANi morphology were associated with a lower sensitivity of −52.38 mV/pH and a slower response time of 46 s. LIG-5.6-150 exhibited the lowest apparent porosity (18.60%), higher sheet resistance (7.43 Ω/sq), structural discontinuities, and more pronounced PANi clustering, which coincided with the lowest sensitivity (−45.22 mV/pH), highest drift rate (2.83 mV/h), and longest response time (53.6 s). LIG-6.8-250 exhibited higher apparent porosity (38.07%) and less pronounced PANi clustering, together with improved sensitivity (−54.4 mV/pH) and a shorter response time (31 s). Its relatively higher sheet resistance (7.12 Ω/sq) was consistent with the observed discontinuities in the LIG network (Figure 13). In addition, variations in EB/ES states, oxidation states, and polaronic character supported by Raman analysis (Section 3.3.4) may affect the variations in pH-sensing performance.^74,75^ These results suggest that laser-processing conditions can be controlled to tune LIG morphology and electrical properties as a transducing layer, thereby influencing PANi deposition and improving pH-sensing performance of PANi/LIG electrodes. Table 5 has been included to summarize the sensing performance of the present PANi/LIG pH sensor in comparison with previously reported flexible and LIG-based potentiometric pH sensors.

**Table 4:** Summary of the pH-Sensing Performance of PANi/LIG pH Sensors

Sensors	Sensitivity (mV/pH)	Stability (mV/h)	Reversibility (mV)	Response time, *t*_90_ (s)
LIG-4.4-150	–52.38	2.5	3.5	46
LIG-5.6-150	–45.22	2.83	3.57	53.6
LIG-6.8-250	–54.4	1.5	3.66	31
LIG-9.2-350	–60.04	1.67	2.06	13.2

**Table 5:** Comparison of the Present PANi/LIG pH Sensor with Recently Reported Flexible and LIG-Based pH Sensors

Sensor material	Substrate	Detection method	Linear pH range	Sensitivity (mV/pH)	Response time (s)	References
PANi/LIG	polyimide	Potentiometric	4–7	–51.5	–	^76^
PANi/CNT	PET	Potentiometric	1–13	–58	–	^77^
PANi nanofiber	PET	Potentiometric	3.9–10.1	–62.4	12.8	^9^
PANi/graphite	PET	Potentiometric	3–10	–53	15	^78^
PANi	Carbon fiber cloth	Potentiometric	4–12	–60.9	–	^79^
PANi/glassy carbon	PVC	Potentiometric	5.13–8.27	–55	–	^80^
PANi/carbon black	–	Potentiometric	3–8	–74	–	^81^
PANi/LIG	polyimide	Potentiometric	4–10	–56.7 ± 2.6	<10	^23^
PANi/MXene/LIG	PDMS	Potentiometric	4–9	–57.03	–	^82^
PANi/LIG-9.2-350	polyimide	Potentiometric	4–9	–60.04	13.2	This work

## 4 Conclusion

This study demonstrates that laser-processing conditions can be controlled to tune the characteristics of LIG electrodes as transducing layers in PANi-based potentiometric pH sensors. Different combinations of laser power and scan speed resulted in changes in LIG morphology, apparent porosity, graphitic structure, and electrical properties, which influenced PANi morphology, coverage, and structural characteristics, and subsequently pH-sensing performance. SEM and EDS analyses showed that highly porous and more continuous and electrically conductive LIG promoted more uniform fibrous PANi deposition, whereas less porous and less continuous LIG led to localized PANi clustering. Raman deconvolution suggested differences in the protonation, oxidation, and polaronic characteristics of PANi deposited on LIG electrodes fabricated under different laser-processing conditions.

Among the PANi/LIG sensors, PANi/LIG-9.2-350 exhibited the best overall sensing performance, with a pH-sensitivity of –60.04 mV/pH, 12 h drift rate of 1.67 mV/h, hysteresis of 2.06 mV, average repeatability RSD of 1.42%, and response time of 13.2 s. This improved performance is attributed to its uniformly distributed nanofibrous PANi coverage without localized clustering, supported by highly porous and continuous LIG network with low sheet resistance. These characteristics may facilitate electrolyte access, increase proton-accessible PANi sites, and promote reversible proton exchange at the PANi/LIG interface. In contrast, sensors with lower pore accessibility, higher sheet resistance, or more pronounced PANi clustering showed reduced sensitivity and slower responses. These results suggest that laser-processing-dependent properties of LIG transducers can be engineered during laser processing to enhance the performance of flexible PANi/LIG pH sensors. Future work will evaluate the PANi/LIG sensor in application-relevant real samples and assess the effects of sample composition and selectivity.

## Supplementary Material



## Notes

Additional experimental details and characterization data, including LIG screening results, cyclic voltammograms, Raman analysis, BET/BJH analysis, and additional pH-sensing performance data (PDF)
