# Supplementary material for: Processing-Tuned Laser-Induced Graphene Transducers for Polyaniline pH Sensors
Source: ACS Omega. 2026 Oct 27;11(44):69061–76. doi: 10.1021/acsomega.6c09091 (PMC13756687; doi:10.1021/acsomega.6c09091)
Supplement: Supplementary file 1 [file ao6c09091_si_001.pdf]

Processing-Tuned Laser-Induced Graphene Transducers for Polyaniline pH Sensors

Atik Saha, Bo Mi Lee*

Department of Mechanical and Aerospace Engineering, Missouri University of Science and Technology, Rolla, Missouri, 65409 USA

Table S1. Sheet resistance of LIG electrodes obtained during the initial screening of laser power and scan speed combinations used to establish the viable LIG processing window.

Sheet resistance ± SEM, (ohm/sq)									
Speed, mm/s<br>Power, W	30	50	100	150	200	250	300	350	400
2	3.72±0.37	5.27±0.27							
3.2	1.34±0.17	2.59±0.48	3.66±0.23	5.97±0.5	15.96±.79				
4.4	1.52±0.23		2.63±0.17	3.44±0.32	5.12±0.4	6.33±0.83	8.73±1.79		
5.6	2.09±0.61			7.43±1.02	8.5±0.6	8.4±0.45	10.17±.3	12.87±1	
6.8					6.95±1.2	7.12±1.24	8.13±.87	8.46±1.25	11.09±0.56
8					6.77±0.69	7.45±0.67	8.55±.85	9.32±1.32	10.86±1.4
9.2				1.59±0.28		2.47±0.57	4.08±.85	3.44±.4	4.58±0.23
10.4							4.89±.89	3.5±.59	11.775±2.2

Table S2. Relative polaron-associated Raman spectral weight of PANi/LIG electrodes determined from the deconvoluted Raman bands.

Sample	Polaron-associated spectral weight (%)
LIG-4.4-150	25.51
LIG-5.6-150	17.3
LIG-6.8-250	21.74
LIG-9.2-350	28.4

Table S3. Mean pH sensitivity ± standard deviation (SD) calculated from independently fitted calibration curves.

Sample	Sensitivity±SD
LIG-4.4-150	–52.38±2.32 mV/pH
LIG-5.6-150	–45.22±6.55 mV/pH
LIG-6.8-250	–54.40±2.63 mV/pH
LIG-9.2-350	–60.04±0.35 mV/pH

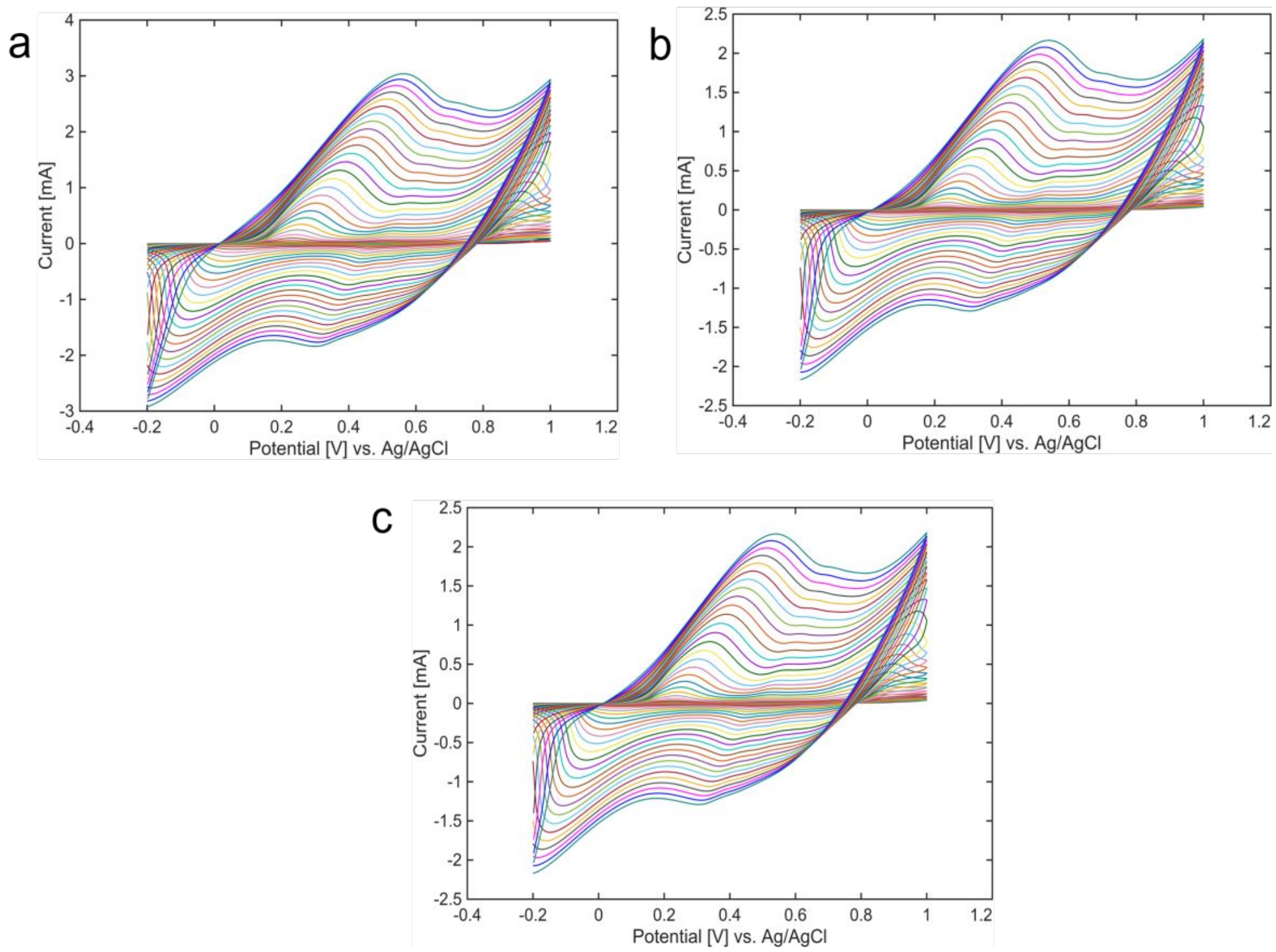

Figure S1. Cyclic voltammograms of PANi electropolymerization on the (a) LIG-5.6-150, (b) LIG-6.8-250, and (c) LIG-9.2-350 electrodes.

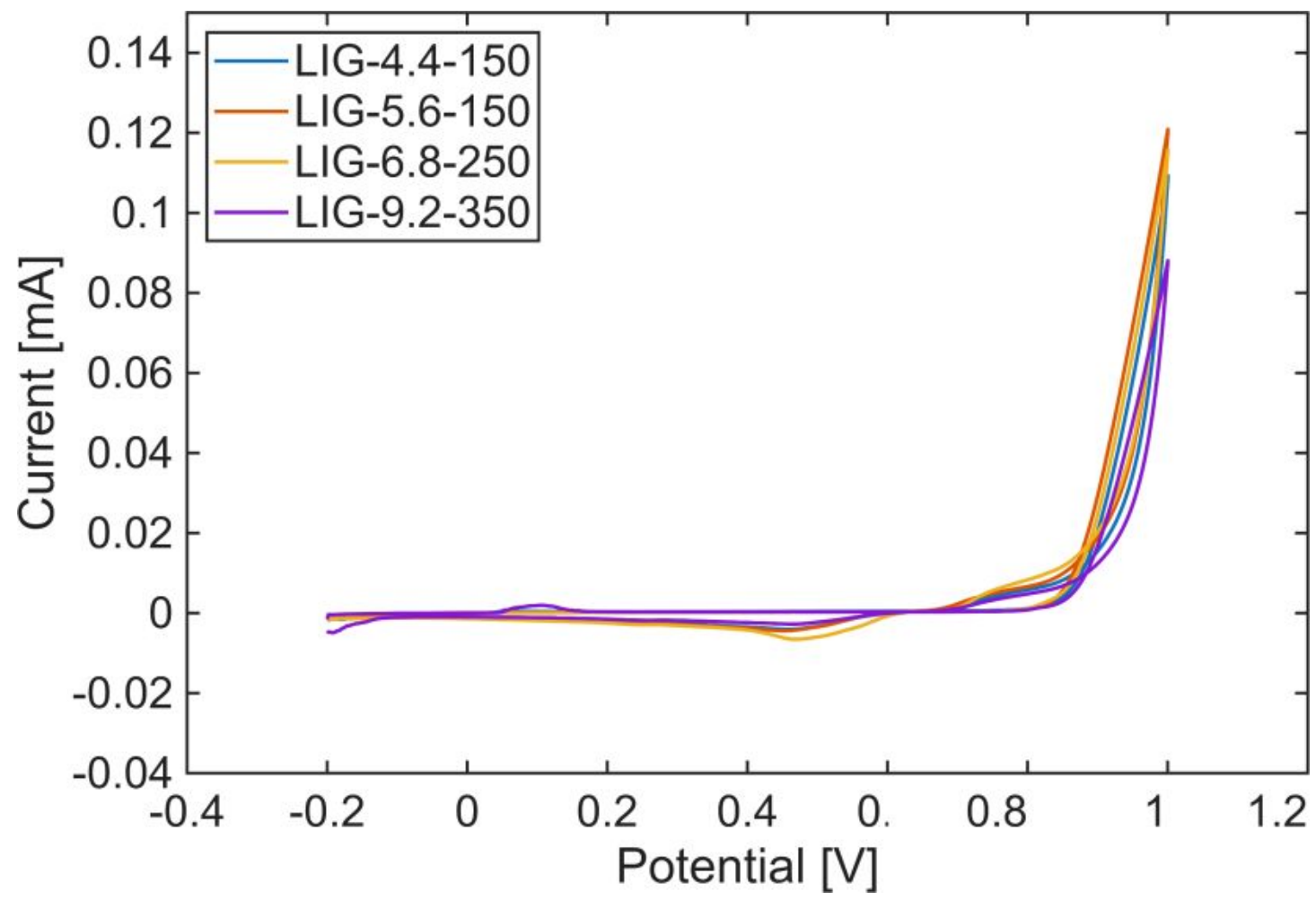

Figure S2. Initial cyclic voltammograms during electropolymerization of PANi on LIG electrodes fabricated under four different laser processing conditions: LIG-4.4-150, LIG-5.6-150, LIG-6.8-250, and LIG-9.2-350.

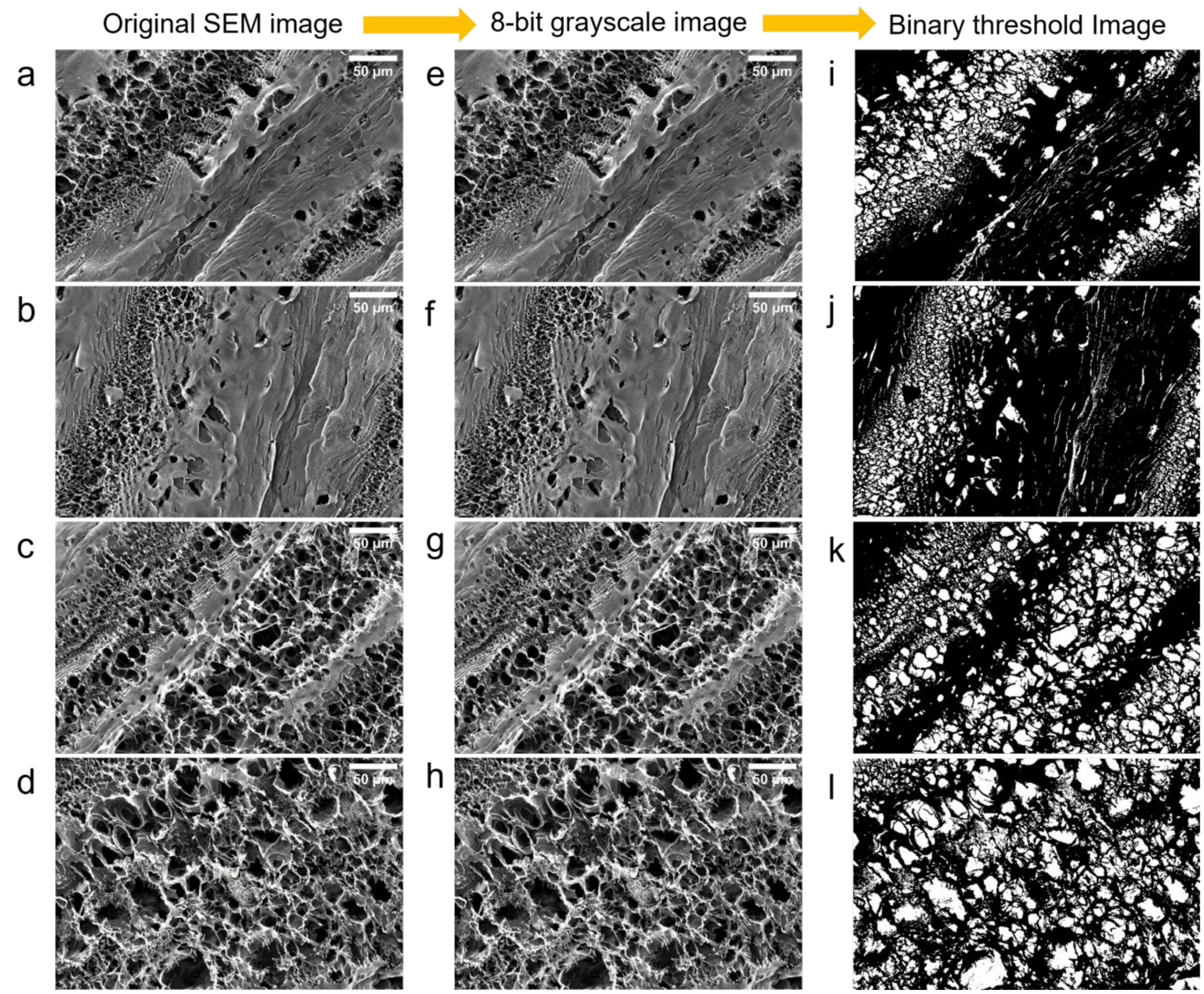

Figure S3. Image-processing workflow used to estimate the apparent surface porosity of LIG electrodes using ImageJ. The original SEM images of LIG-4.4-150, LIG-5.6-150, LIG-6.8-250, and LIG-9.2-350 are shown in (a–d), respectively. The corresponding 8-bit grayscale images are shown in (e–h), followed by thresholded binary images in (i–l). In the binary images, the white regions indicate pore openings, whereas the black regions represent the solid LIG surface.

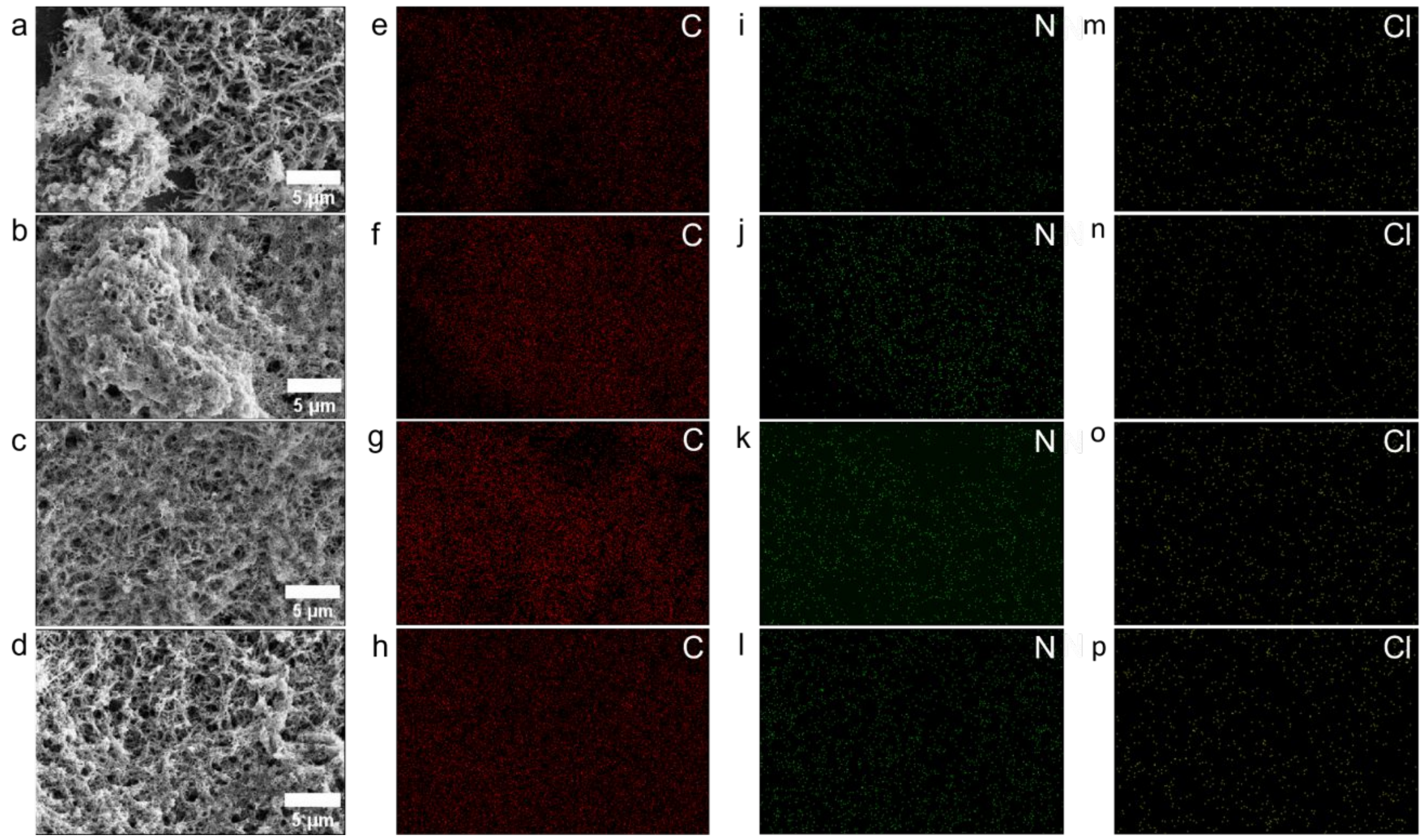

Figure S4. SEM micrographs and corresponding EDS elemental maps of the PANi/LIG electrodes. The SEM micrographs of PANi/LIG-4.4-150, PANi/LIG-5.6-150, PANi/LIG-6.8-250, and PANi/LIG-9.2-350 are shown in (a–d), respectively. The corresponding C elemental maps are shown in (e–h), the N elemental maps in (i–l), and the Cl elemental maps in (m–p). The elemental maps demonstrate the distribution of carbon, nitrogen, and chlorine across the PANi/LIG surface.

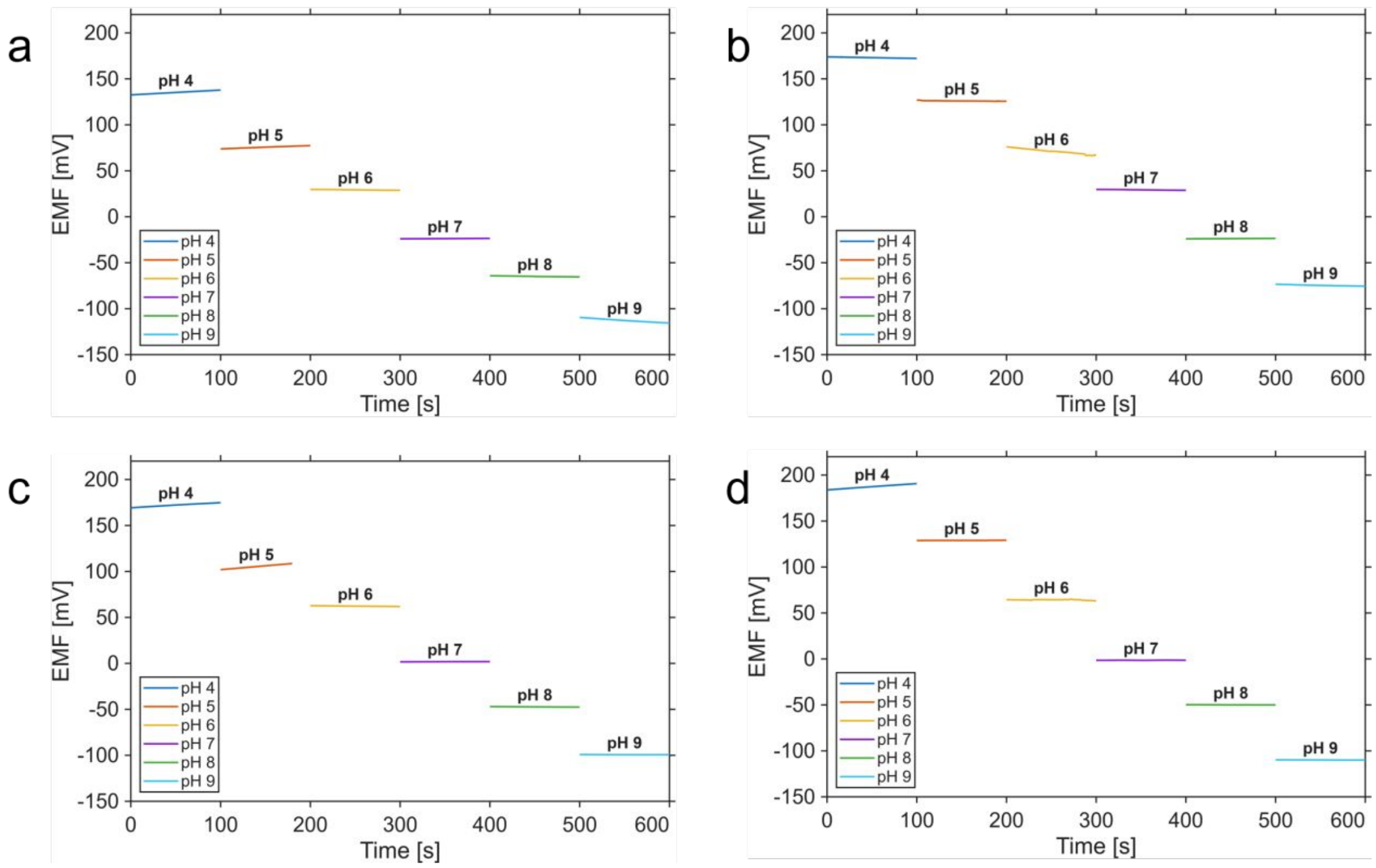

Figure S5. Time-dependent potentiometric EMF responses of PANi/LIG pH sensors measured in buffer solutions from pH 4 to 9: (a) LIG-4.4-150, (b) LIG-5.6-150, (c) LIG-6.8-250, and (d) LIG-9.2-350.
